# Wireless wearable potentiometric sensor for simultaneous determination of pH, sodium and potassium in human sweat

**DOI:** 10.1038/s41598-024-62236-3

**Published:** 2024-05-21

**Authors:** Nahid Rezvani Jalal, Tayyebeh Madrakian, Mazaher Ahmadi, Abbas Afkhami, Sina Khalili, Morteza Bahrami, Majid Roshanaei

**Affiliations:** 1 Department of Analytical Chemistry, Faculty of Chemistry and Petroleum Sciences, Bu-Ali Sina University, Hamedan, 6517838695 Iran; 2 Department of Computer Engineering, Faculty of Engineering, Bu-Ali Sina University, Hamedan, 6517838695 Iran; 3 Biomedical Engineering Department, School of Electrical Engineering, Iran University of Science and Technology, Tehran, 1684613114 Iran

**Keywords:** Wearable potentiometric sensor, Sweat analysis, Polyaniline, Prussian blue analogues, Manganese oxide, Ion-selective electrode, Sensors, Health care, Electrochemistry

## Abstract

This paper reports on the development of a flexible-wearable potentiometric sensor for real-time monitoring of sodium ion (Na^+^), potassium ion (K^+^), and pH in human sweat. Na_0.44_MnO_2_, polyaniline, and K_2_Co[Fe(CN)_6_] were used as sensing materials for Na^+^, H^+^ and K^+^ monitoring, respectively. The simultaneous potentiometric Na^+^, K^+^, and pH sensing were carried out by the developed sensor, which enables signal collection and transmission in real-time to the smartphone via a Wi-Fi access point. Then, the potentiometric responses were evaluated by a designed android application. Na^+^, K^+^, and pH sensors illustrated high sensitivity (59.7 ± 0.8 mV/decade for Na^+^, 57.8 ± 0.9 mV/decade for K^+^, and 54.7 ± 0.6 mV/pH for pH), excellent stability, and good batch-to-batch reproducibility. The results of on-body experiments demonstrated that the proposed platform is capable of real-time monitoring of the investigated ions.

## Introduction

Accessing blood as a biofluid poses a significant challenge because some approaches require breaching the skin surface to access the sample, which is an invasive method, despite some innovate non-invasive methods such as magnetohydrodynamics^1^. Sweat is a non-invasively accessible biofluid that contains a variety of biological, and chemical markers associated with human health, such as proteins, metabolites, and electrolytes^2–4^. The sodium ion (Na^+^), potassium ion (K^+^), and pH are of particular interest among biomarkers of sweat due to their metabolism in the body. Therefore, the level of Na^+^, K^+^, and pH in sweat is an effective marker of a variety of physiological conditions.

The majority of sweat electrolytes are Na^+^ ions and K^+^ ions, which can offer vital information on physical and mental well-being^5^. Excessive sodium loss through perspiration results in severe hyponatremia and low sodium concentrations in the blood (< 135 mM), which can have a negative impact on physical and mental activities. Furthermore, abnormal sweat Na^+^ levels are clinically significant for diagnosing cystic fibrosis, which is caused by a failure in Cl^-^ reabsorption at eccrine glands. In addition, low potassium levels (< 3.5 mM) cause muscle fatigue, cramping, paralysis, heart arrhythmia, and even severe cardiac failure^6^. Also, human sweat pH is an important indicator of skin illnesses, diabetes, and cystic fibrosis^7,8^. Dermatitis and fungal infections on the skin, for example, can cause a change in sweat pH^9^. Diabetics' sweat has acidic pH, while cystic fibrosis patients' sweat frequently has a basic pH level^10,11^. Therefore, real-time monitoring of Na^+^, K^+^, and pH in sweat is necessary to offer important data for clinical diagnosis.

Sweat evaluation includes numerous stages consisting of sweat collection, extraction, storage, and detection. since sweat composition relies closely on its generated position and type, the sampling technique can affect the sweat evaluation consequences^12^. Traditional sweat sampling is not capable of in situ and real-time monitoring and might cause numerous problems including sample evaporation, degradation, or infection. Emerging wearable sensors, which are typical of skin-compatible devices, enable sweat continuous monitoring in wearable formats in the body^13^. These sensors act as a “lab on the pores and skin” capable of sample collection, storage, and measurement, specifically appropriate for noninvasive, and real-time monitoring of biomarkers in sweat^14^. Therefore, in situ sampling of sweat is of particular interest for great importance for clinical diagnosis^15^.

Today, wearable gadgets allow for the digitalization of a broad range of parameters from the human body^16^. Wearable electrochemical sensors are widely used in wearable sensing as they are the ideal transducer from the biochemical domain to the digital domain which can used in data treatment^17^. Electrochemical sensors are the best choice for wearable sweat sensors due to their high performance, low cost, and wide applicability^18^. Wearable potentiometric ion sensors among electrochemical sensors getting more popular attention for real-time ion monitoring in sweat samples owing to the following properties: (i) simplicity, owing to the portability of the miniaturized and non-invasive sensors; (ii) analytical and mechanical robustness during the individual's activity. Wearable potentiometric ion sensors are an exciting analytical platform that combines chemical, material, and electronic efforts to supply physiological information during certain human activities^19^.

Mazzaracchio et al. presented an ion-selective carbon black (CB) for sensing Na^+^ ion in a potentiometric sensor^20^. The graphite ink as a working electrode and ink as a pseudo-reference electrode Silver/silver chloride were successfully printed on the substrate. Then pseudo-reference electrode and working electrode were modified by drop casting of reference membrane solution (PVB + NaCl) and ion-selective membrane cocktail containing CB, respectively. The sensor array integrated with a portable wireless potentiometer was fabricated to detect K^+^, Na^+^, and Ca^2+^ in real urine and simulated sweat samples, by Teekayupak et al.^21^. This sensor array shows detection limits 10^–5^ M, 10^–5^ M, and 10^–4^ M for the detection of K^+^, Na^+^, and Ca^2+^ ions, respectively. Hui et al. introduced an Au nanoparticle/siloxene-based solid contact (SC) supported by a substrate made of laser-inscribed graphene on poly(dimethylsiloxane) for the noninvasive detection of sweat Na^+^ and K^+^. These SC nanocomposites prevent the formation of a water layer during ion-to-electron transfer, preserving 3 and 5 μV/h potential drift for the Na^+^ and K^+^ ion-selective electrodes, respectively, after 13 h of exposure. Yet in the real-time sweat analysis, some key challenges exist such as the periodic generation of sweat, the capture rate of sweat, the mixing of the produced sweat at various times, asymmetrical transport of sweat over the skin, inconvenient evaporation of sweat, and disordered volume of sweat^6^. Emerging microfluidic technology with miniaturization capability is capable of the sampling, collection, and analysis of sweat^22^_ENREF_21_ENREF_21. The design and introduction of suitable ionophores as the sensing material for wearable potentiometric ion sensors is the most important part of their development. In 2022, our group introduced Na_0.44_MnO_2_ as a sensing material for the detection of Na^+^ in sweat by using a wearable potentiometric sensor^23^. Also, polyaniline (PANI) as a conductive polymer is a popular material for the determination of pH in sweat due to its H-doped properties^5^. Given the polymerization mechanism of PANI, which is a proton-based polymer, it can get or lose proton during redox reactions. Therefore, the H-doing property of PANI leads to it being sensitive to H^+^ ion^24^.

Prussian blue analogues (PBAs) containing alkali metal cations as counter ions can be used to fabricate ion-selective electrodes in the potentiometric sensors due to the ability to reversibly incorporate alkali metal cations^25,26^. In addition, PBAs are particularly attractive materials for the fabrication of K-ion batteries^27,28^. Mortimer et al. introduced K_2_Ni[Fe(CN)_6_] as a PBAs useful material for the sensing of K^+^ in a potentiometric sensor. The K_2_Ni[Fe(CN)_6_] was synthesized by electrochemical method on the glassy carbon electrode^25^. Giorgetti et al. synthesized the KNiFe(CN)_6_ and the potentiometric response of the proposed sensor for K^+^ in the presence of other cations was studied^26^. However, the employment of this material in wearable potentiometric applications is limited. These results sparked the use of K-mediated Co–Fe-based PBAs architectures (K_2_Co[Fe(CN)_6_]) as an attractive material to fabricate a wearable potentiometric sensor for real-time monitoring of K^+^ in sweat. According to the above-mentioned, K_2_Co[Fe(CN)_6_] as a K-rich PBAs can be capable of reversibly incorporating K^+^ and its potentiometric response toward K^+^ can be investigated.

Here, we developed a flexible-wearable potentiometric sensor for simultaneous real-time monitoring of Na^+^, K^+^, and pH in sweat. This study aims to introduce a K_2_Co[Fe(CN)_6_] as an attractive sensing material (K^+^-ionophore) for monitoring K^+^ and designing a microfluidic platform in a wearable potentiometric sensor. The array of electrodes and microfluidic channels were defined by using of sputtering technique. The Ag/AgCl/polyvinyl butyral (denoted as Ag/AgCl/PVB) as a quasi-reference electrode was synthesized on the reference electrode area. The working electrode surface was coated by PANI, Na_0.44_MnO_2_, and K_2_Co[Fe(CN)_6_] were used as sensing materials for monitoring Na^+^, K^+^, and pH in sweat. The paper strip was used as a fluidic channel for sweat real-time transport. Embedding the paper strip between the flexible electrodes allows better sampling for constant sweat flow and prevents sweat from evaporating. Then, the electrode platforms were connected to the miniature printed circuit board (PCB), which could enable multiplex decoding of sweat and the wireless transmission of signals to the host smartphone. Finally, the proposed microfluidic device can able to real-time monitor the pH, Na^+^, and K^+^ as useful markers by consuming minimal sweat volume during exercise activities.

## Experimental

### Ethics

The Bu-Ali Sina University Research Ethics Committee has approved the experiments and all experiments were performed in accordance with relevant guidelines and regulations. Informed consent was obtained from the human participant to publish their information/images.

### Chemicals and apparatus

The vendor information of chemicals and apparatus have been included in the supporting information file. Artificial sweat was prepared by mixing of KCl (3 mM), NaCl (10 mM), NH_4_Cl (3 mM), CaCl_2_ (0.4 mM), Mg(NO_3_)_2_. 6 H_2_O (50 μM), urea (22 mM), glucose (20 mM), lactic acid (25 mM), and uric acid (25 μM) in deionized water^29^. Phosphate buffer solution (PBS) was prepared by mixing appropriate amounts of NaH_2_PO_4_ /Na_2_HPO_4_ salts with 0.1 M ionic strengths in deionized water.

ESP32 with a built-in Wi-Fi board was used as the microcontroller unit for real-time collecting, processing, and transferring of data to the host smartphone. The PCB microcontroller was powered by a 3.7-V lithium rechargeable battery assembled into a battery module (T6845-C model). Also, the readout potential of the PCB microcontroller was calibrated by a digital multimeter (Victor, 88C model), and commercial power supply (TPS-3010U model). The box of the potentiometric sensor platform was fabricated by 3D printers (Author L Pro, Iran). Whatman grade 2 filter paper, supplied by Whatman GE Healthcare (U.S.) was used as a paper strip. It should be noted that the filter paper with 0.18 mm thickness was washed with deionized water and dried in room ambient to eliminate pollutants and dust. Piton crystal adhesive tape (5cm width, Iran) was used as a non-conductive insulator. The commercial PVC thin sheet (210 mm length, 297 mm width, and 0.1 mm thickness) serves as a platform for a potentiometric sensor (Uxcell, China). It is worth mentioning that several platforms (40 mm × 30 mm) for potentiometric sensors are obtained from this PVC sheet. The laser instrument (Rotec, Iran) with 80 W of power tube and working table 60 cm × 40 cm was used for the design and define electrodes platform. The work table is the area inside the machine where the PVC sheet is placed on it for cutting. The sputtering instrument (Epsilon, Iran) with copper and silver targets was employed to coat electrode substrates with copper nanoparticles (Cu NPs) and silver nanoparticles (Ag NPs) at 350 V and 470 V, respectively. The reference electrode substrate was coated with layers of Cu NPs as a sacrificial layer and followed by coating with layers of Ag NPs to increase both adhesion and electrical conductivity. However, the ion-selective electrode (ISE) substrates only were coated by the Cu NPs layer.

### Preparation of Na^+^, K^+^, and pH selective membranes

The methods used for the synthesis of PANI, Na_0.44_MnO_2_, and K_2_Co[Fe(CN)_6_] have been included in supporting information file. The cocktail of Na^+^-selective membrane was prepared by mixing 8 wt % of Na^+^ ionophore (i.e. Na_0.44_MnO_2_), NaTPB (2 wt %), PVC (30 wt %), and DBP (60 wt %). The cocktail of K^+^-selective membrane consisted of K_2_Co[Fe(CN)_6_] (9 wt %) as a K^+^ ionophore, NaTPB (2 wt %), PVC (29 wt %), and DBP (60 wt %). To make the membrane solution, 200 mg of the corresponding cocktails was dissolved in 1 mL of THF. Also, the pH sensing membrane was composed of 20 mg of PANI as an optimum amount in 2 mL of THF. More detailed information about optimizing the membrane composition is listed in Tables S1-S3.

### Fabrication of wearable potentiometric sensor platform

**Figure 1:**
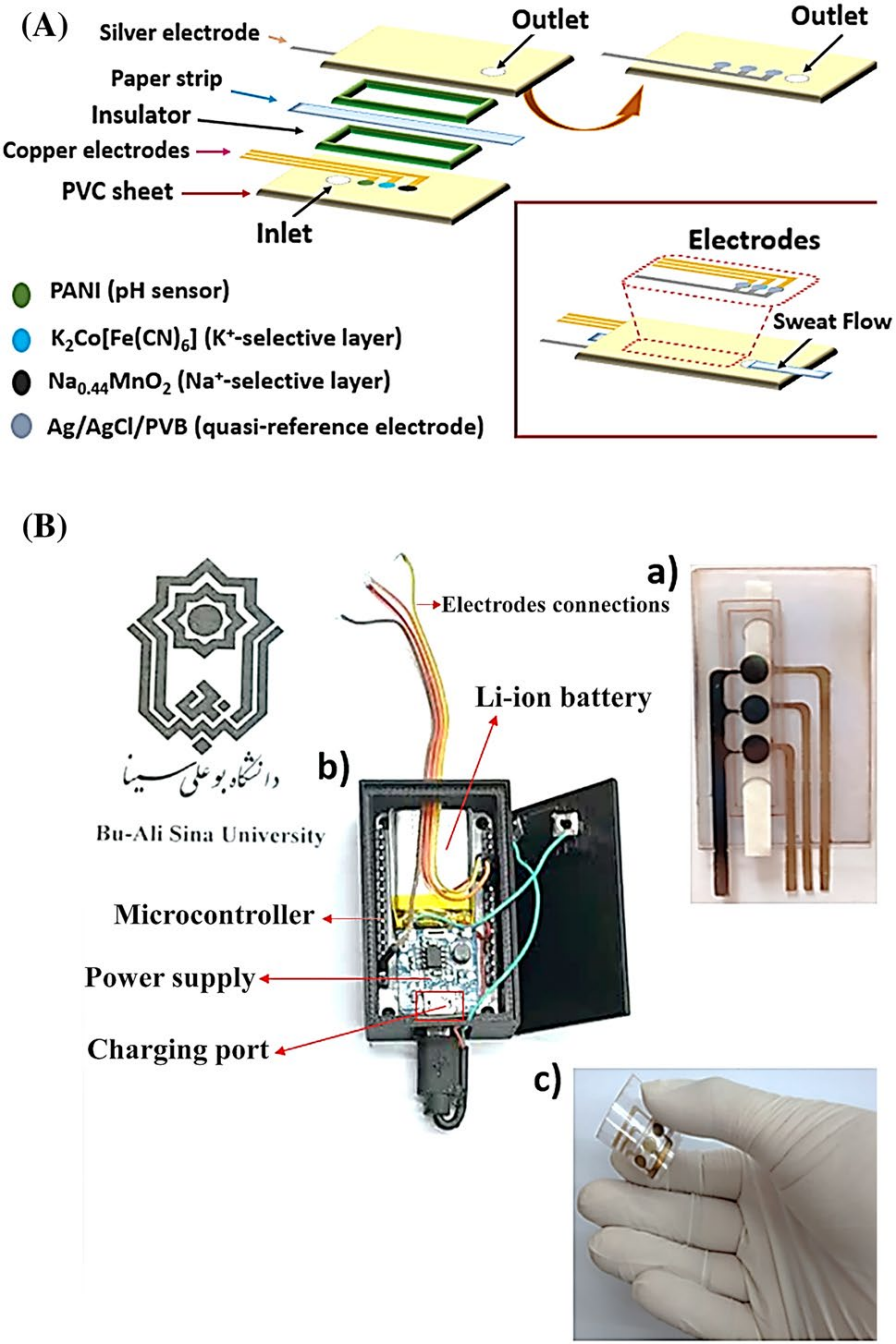
(**A**) Schematic illustration of the wearable potentiometric sensor; (**B**) photograph of the potentiometric sensing platform (**a**), photograph of the potentiometric sensor box containing the PCB and Li-ion battery (**b**), and flexibility of the potentiometric sensing platform (**c**).

The fabrication process of the wearable potentiometric sensor platform is illustrated in Fig. 1A. An array of electrodes containing three working electrodes with a geometric area of 0.2 cm^2^ were printed on the flexible PVC thin sheet. Subsequently, the layers of Cu NPs were deposited onto the specific electrode areas by sputtering with the help of the additional non-conductive mask. Then, ion-selective membranes onto the corresponding electrode area were prepared by drop-casting 10 μL of the Na^+^-selective membrane cocktail, 10 μL of the K^+^-selective membrane cocktail, and 10 μL of the pH-sensing membrane cocktail. The quasi-reference electrode pattern was defined on the thin PVC sheet through a laser cutter and then coated with 0.37 µm and 0.55 µm of Cu NPs and Ag NPs, respectively through sputtering with the help of the additional non-conductive mask. The Ag/AgCl/PVB was prepared to the method reported in the literature^29^. 5 μL of FeCl_3_ solution (0.1 M) is drop-casted onto the surface of the reference electrode coated with Cu NPs and Ag NPs and allowed to chlorinate for 2 min. Following that, the electrode is rinsed with deionized water. Hence, Ag/AgCl layers are prepared on the electrode surface. 10 μL of PVB mixture containing 79.1 mg of PVB and 50 mg of NaCl dissolved in 1 mL of methanol was dropped on top of the Ag/AgCl layers. The fabricated quasi-reference electrode is denoted as Ag/AgCl/PVB. After that, the quasi-reference electrode pattern printed on a thin PVC sheet was placed in front of the working electrode patterns printed on a thin PVC sheet. To prevent the electrical connection between the printed quasi-reference electrode pattern and the printed working electrode patterns, two non-conductive insulators were used to separate them. The optical images of electrodes and spacers are shown in Figure S1. Next, a washed paper strip was employed for passing the sweat sample between two thin PVC sheets. Finally, all layers were pressed with commercial flat steel with internal electrical heating to hinder the leakage of the sweat sample. Therefore, the sweat sample can move on the paper by capillarity properties. Figure 1B shows optical images of the potentiometric sensor platform and its accessory box. The above view of the accessory box of the potentiometric sensor is shown in Figure S2. The chemicals used to make the sensor are not toxic or hazardous and in addition, its design prevents the direct attachment of the surface of modified electrodes to the skin. Also, the thin PVC sheet, which was purchased commercially, is in contact with the skin and is completely non-toxic and non-hazardous.

### Potentiometric measurements

The performance of PANI, Na_0.44_MnO_2_, and K_2_Co[Fe(CN)_6_], towards sensing of pH, Na^+^, and K^+^, respectively was investigated by modification of single-printed working electrode pattern surface by pH selective membrane, Na^+^-selective membrane, and K^+^-selective membrane with the different mass percentages of nanostructured PANI, Na_0.44_MnO_2_, and K_2_Co[Fe(CN)_6_]. The modified single-printed electrode and a printed Ag/AgCl/PVB electrode were used as a working electrode and quasi-reference electrode, respectively. The modified working electrode and reference electrode were immersed in an aqueous solution, and the EMF was measured continuously while the concentration of H^+^, Na^+^, and K^+^ in the standard solutions was changed. Then, the calibration slope as the analytical response was calculated. pH sensor was tested in PBS (0.1 M) with pH ranging from 4.0 to 8.0, and selectivity was evaluated in the presence of different ions of Ca^2+^, NH_4_^+^, Mg^2+^, K^+^, and Na^+^ in a PBS at pH 6.0. Also, Na^+^ and K^+^ sensors were studied in concentrations ranging from 1 to 160 mM of NaCl solution, and 0.5 mM to 80 mM of KCl solution, respectively. Then, the selectivity of the Na^+^ and K^+^ sensors was investigated by the subsequent addition of Ca^2+^, NH_4_^+^, Mg^2+^, Na^+^, and K^+^ into a 15 mM of Na^+^ solution, and 5 mM K^+^ solutions, respectively.

Furthermore, the performance of the proposed three sensors integrated into the platform was evaluated in the out-body configuration. A PCB microcontroller was connected to the proposed sensors, which could record pH, Na^+^, and K^+^ levels as wireless in solution. Next, the pre-fabricated potentiometric sensor was immersed in deionized water. After that, the different concentrations were pipetted to the artificial sweat, and the analytical signals were recorded (Supporting video). A smart armband for the on-body test was designed and fabricated with a PCB microcontroller for real-time monitoring of Na^+^, K^+^, and pH levels in human sweat during exercise activity.

## Results and discussion

### Characterization of PANI, Na_0.44_MnO_2_, and K_2_Co[Fe(CN)_6_]

**Figure 2:**
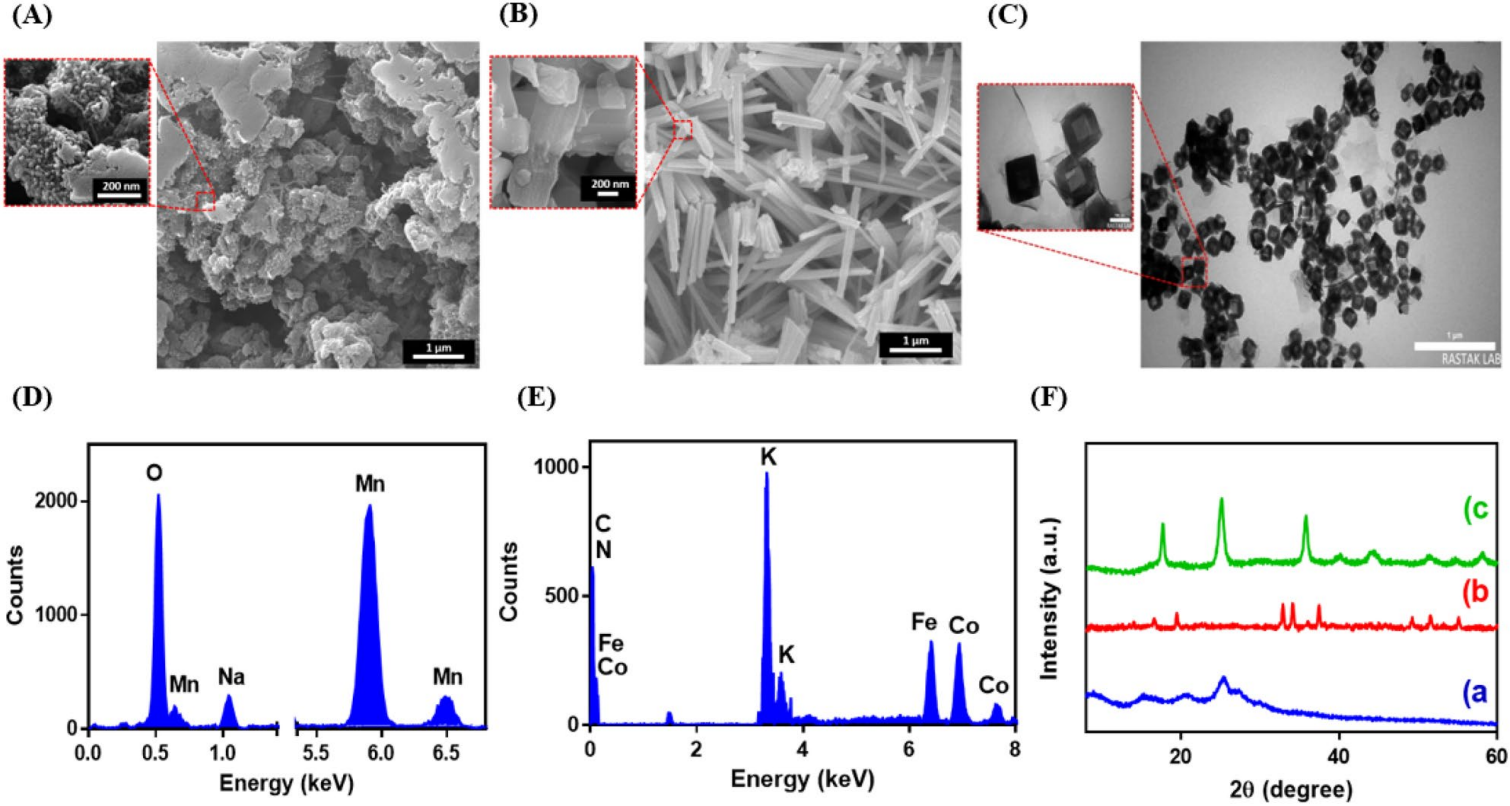
FESEM images of (**A**) PANI, (**B**) Na_0.44_MnO_2_; TEM image of (**C**) K_2_Co[Fe(CN)_6_]; EDX spectra of (**D**) Na_0.44_MnO_2_ and (**E**) K_2_Co[Fe(CN)_6_]; (**F**) XRD patterns of (**a**) PANI, (**b**) Na_0.44_MnO_2_, and (**c**) K_2_Co[Fe(CN)_6_].

The FESEM technique was performed to investigate the morphology of PANI, and Na_0.44_MnO_2_ and TEM technique was used to study the morphology of K_2_Co[Fe(CN)_6_]. Figure 2A–C display FESEM images of PANI, Na_0.44_MnO_2_, and TEM images of K_2_Co[Fe(CN)_6_], respectively. As shown in Fig. 2A, the FESEM of PANI demonstrates a random interconnected network and flat plate structure^30^. The FESEM of Na_0.44_MnO_2_ powder displays anisotropic nanoparticles with rod-like shapes (Fig. 2B). The morphology of synthesized Na_0.44_MnO_2_ was similar to the FESEM images reported previously^31^. Figure 2C shows the box morphology of K_2_Co[Fe(CN)_6_] with an average size of around 145 nm^32^. The small size of K_2_Co[Fe(CN)_6_] leads to an increase in contact with a solution containing K + ions, which is desirable for the ion transport of K^+^^28^.

Also, the elemental analysis of Na_0.44_MnO_2_ and K_2_Co[Fe(CN)_6_] was characterized by EDX. The EDX spectrum of the Na_0.44_MnO_2_ indicates only Na, Mn, and O elements with a weight ratio of 11.81%, 29.51%, and 58.68%, respectively (Fig. 2D). The Na/Mn and O/Mn ratios were evaluated by EDX. The obtained atomic ratios for Na/Mn and O/Mn were calculated at 0.40, and 1.98, which was approximately consistent with the theoretical formula of Na_0.44_MnO_2_. As shown in Fig. 2E, the EDX spectrum verifies the presence of K, Fe, and Co elements with weight percent of 13.98, 7.57, and 7.72%, respectively. Also, the EDX result of K_2_Co[Fe(CN)_6_] shows the atomic ratio of K/Co/Fe is 1.81: 1: 0.98, which was approximately consistent with the theoretical formula.

The XRD analysis was utilized to study PANI, Na_0.44_MnO_2_, and K_2_Co[Fe(CN)_6_] crystalline compounds (Fig. 2F). The PANI shows characteristic diffraction peaks at 2θ position of 9.5° (0 0 1), 14.9° (0 1 1), 21.3° (1 0 0), 25.5° (1 1 0), and 27.5° (1 1 1), confirming the formation of PANI (Fig. 2F, a)^33^. As shown in Fig. 2F, b, the XRD of Na_0.44_MnO_2_ shows distinctive diffraction peaks at 2θ position of 16.6° (1 4 0), 19.5° (2 0 0), 32.8° (0 8 0), 34.3° (3 4 0), 36.1° (3 5 0), 37.6° (3 6 0), 49.4° (2 9 1), and 51.6° (4 0 1) related to the orthorhombic lattice structure of Na_0.44_MnO_2_ with a lattice constant of a = 8.9050 Å, b = 2.8520 Å, c = 11.0820 Å, α = 90.00°, β = 90.00°, and γ = 90.00° (JCPDS 96–152-8294)^31^. The XRD pattern of K_2_Co[Fe(CN)_6_] exhibits sharp reflection peaks which appeared at the 2θ position of 17.6° (2 0 0), 25.1° (2 2 0), 30.7° (3 1 1), 35.7° (4 0 0), 40.0° (4 2 0), 44.1° (4 2 2), 51.4° (4 4 0), and 58.1° (6 2 0), attributed to the face-centered cubic lattice structure of K_2_Co[Fe(CN)_6_] with a lattice constant of a = 10.0800 Å, b = 10.0800 Å, c = 10.0800 Å, α = 90.00°, β = 90.00°, and γ = 90.00° (JCPDS 01–075-0038)^32^ (Fig. 2F, c). The obtained results confirm that the PANI, Na_0.44_MnO_2_, and K_2_Co[Fe(CN)_6_] compounds were successfully synthesized.

Figure S3A shows the Ag NPs layers deposited on the PVC sheet which has a sphere morphology in the nanometer size. However, there are cracks in the deposited layer that reduce the stability of the Ag NPs layers. Hence, the Ag NPs were deposited on the PVC Sheets coated with Cu NPs to increase the stability of the coating film. The FESEM image of the Cu NPs layers deposited on the PVC sheet with an average size of around 70 nm is shown in Figure S3B.

Figure S4 displays FESEM images of the Ag NPs layers, Ag/AgCl layers, and Ag/AgCl/PVB layers on the PVC Sheets coated with Cu NPs related to the quasi-reference electrode. Figure S4A shows the morphology of Ag NPs layers with an average size of around 45 nm on the Cu NPs layers. The morphology of the Ag NPs layers was changed when they converted to Ag/AgCl during the chlorination process. The same result was also observed by Moya^34^. The nanocube-like and plate-like morphology of the AgCl layer with very sharp corners and clear edges on the Ag NPs layers is displayed in Figure S4B. The FESEM of Ag/AgCl/PVB shows a polymer matrix on the Ag/AgCl layers, while some of AgCl microcrystals with nanocube-like and plate-like morphology are still observed on the surface (Figure S4C). These observations indicated that the Ag/AgCl/PVB as a quasi-reference electrode was successfully formed.

The cross-section views of thin PVC sheet-coated with Cu NPs, Ag NPs/Cu NPs, fabricated AgCl on the Cu NPs/Ag NPs, and PVB coated on the Cu NPs/Ag NPs/AgCl with various thicknesses are displayed in Figure S5. According to Figures S5A and B, the Cu NPs and Ag NPs layers were coated on the PVC sheet with thicknesses of 0.37 µm and 0.55 µm, respectively. Then, 31% of the initial coated Ag NPs layer on the Cu NPs is converted to AgCl, which has an average thickness of 1.2 μm (Figure S5C). The cross-section view of PVB coated on the Cu NPs/Ag NPs/AgCl shows a layer with a thickness of 0.8 μm (denoted as Ag/AgCl/PVB) (Figure S5D).

### Potential behavior of Ag/AgCl/PVB quasi-reference electrode

The chronopotentiometry and cyclic voltammetry (CV) techniques were employed to study the potential behavior of the reference electrode. The purpose of using an Ag/AgCl/PVB electrode instead Ag/AgCl as a quasi-reference electrode lies in the fact that the potential of the Ag/AgCl/PVB electrode was virtually undisturbed by changes in Cl^−^ concentration. This selection would be especially important for signal stabilization in the biofluid environment. As shown in Figure S6A, the potentiometric responses of Ag/AgCl and Ag/AgCl/PVB electrodes in the presence of 2, 8, and 12 mM of NaCl were plotted. The potential of the Ag/AgCl/PVB electrode was undisturbed by changes in Cl^−^ concentration, while the potential of the Ag/AgCl electrode was distracted by changes in Cl^−^ concentration. Also, a well-defined CV curve for 0.1 M KCl and 5 mM of [Fe(CN)_6_]^3−/4−^ vs. Ag/AgCl/PVB was observed at the bare GCE (Figure S6B).

### Sensing performance evaluation

The performance of a potentiometric sensor depends on the membrane composition. The composition and potential responses of Na^+^, K^+^, and pH selective membranes toward goals analytes are listed in Tables S1-S3. The amount of sensing material has a significant impact on the performance of potentiometric sensors. Loading a certain amount of ionophores as a sensing material can determine the interaction of ions with the corresponding ionophore and has a very important effect on sensor sensitivity. The effect of the amount of ionophore on potentiometric sensor performance was studied by modification of ISEs with various amounts of ionophores. The obtained results indicated that the employed ionophore has a significant effect on the potential response of the target sensor. According to tables S1, S2 and S3, the slopes obtained for the amount of 8% for Na^+^ ionophore (Na_0.44_MnO_2_), 9% for K^+^ ionophore (K_2_Co[Fe(CN)_6_]), and 20 mg for H^+^ ionophore (PANI) are very close to the 59.2 mV/Decade value. However, the sensors show a super-Nernstian behavior by increasing the amount of ionophores. The super-Nernstian response of ISEs to Na^+^, K^+^, and H^+^ ions after the optimum membrane is the result of the increase in the concentration of free ionophore, and the concomitant decrease in the concentration of free Na^+^, K^+^, and H^+^ ions in the aqueous boundary layer. It is worth mentioning that the super-Nernstian electrode responses were only observed when the membrane composition optimization was investigated. While the modified electrode responses in optimal conditions completely had the Nernstian behavior.

The nature of the plasticizer is one of the most important parameters that should be considered in the membrane construction of Na^+^, and K^+^ ions^35^. Among the NB, DOP, and DBP as plasticizers, DBP demonstrates the best Nernstian response because it has the moderate dielectric constant (ε). It seems a stronger interaction can create between the univalent ions and the plasticizer. Furthermore, since NB (ε = 36.1) is more polar than DBP (ε = 6.4), it is able to decrease the performance of the sensor due to their tendency to extract other interfering ions. Also, DOP (ε = 5.1) and OA have the lowest polarity and non-polar nature, respectively, and can interact with other interfering ions, resulting in the performance of the sensor being decreased^36,37^. Therefore, DBP was selected as the optimum plasticizer. Also, the slopes observed with different amounts of ionic additive for Na^+^, and K^+^ membranes showed that the closest slope to 59.2 mV/Decade is the membrane with 2% of the ionic additive. The optimized membrane composition for sensing Na^+^, K^+^, and pH levels was highlighted in Tables S1-S3.

Figure S7 shows the potentiometric response of the Na^+^, K^+^, and pH sensors in the 15 mM Na^+^ solution, 10 mM K^+^, and PBS (pH = 6.0) at different temperatures. The potentiometric response of the Na^+^ sensor showed high stability with only a negligible average RSD of 0.9% (Figure S7, a). Similarly, K^+^ and pH sensors displayed good stability with average RSDs of 1.5% and 1.6%, respectively (Figure S7, b, and c). The obtained results indicate that the temperature does not have a significant effect on the potential response of the sensors.

Under optimum condition, the performance of the proposed potentiometric sensor was investigated by different concentrations of H^+^, Na^+^, and K^+^ ions. The PANI was used as an ion–electron transducer for the H^+^ ions. Also, Na-TPB in the membrane of the Na^+^ and K^+^ sensor was utilized as an ion exchanger for the Na^+^ and K^+^ ions for the achievement of stable potential signals.

**Figure 3:**
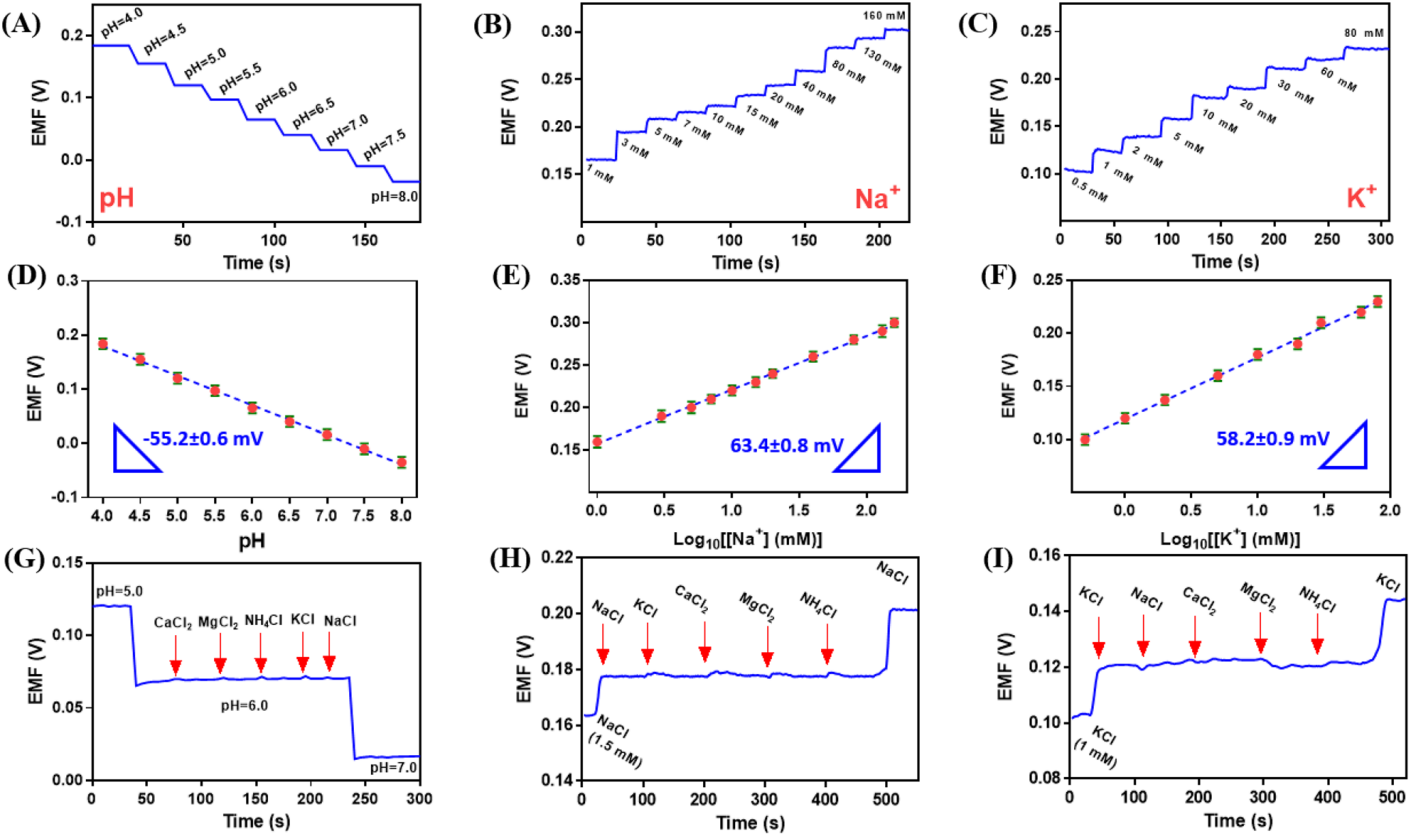
Open-circuit potential responses of the (**A**) pH, (**B**) Na^+^, and (**C**) K^+^ sensors in PBS, NaCl, and KCl solutions by potentiostat/galvanostat Autolab, respectively. Calibration curves for the (**D**) pH, (**E**) Na^+^, and (**F**) K^+^ sensors. Results of the electrode selectivity test for the (**G**) pH, (**H**) Na^+^, and (**l**) K^+^ sensors in complex solutions.

Figures 3A − C show the potentiometric response of the pH sensor in PBS (0.1 M) with pH ranging from 4.0 to 8.0, the Na^+^ sensor in 1 − 160 mM NaCl solution, and the K^+^ sensor in 0.5 − 80 mM KCl solution, respectively. The response of three ion-selective electrodes displays an excellent linear relationship with the change in H^+^, Na^+^, and K^+^ ions levels with sensitivities of 55.2 ± 0.6 mV (R^2^ = 0.9980) for H^+^, 63.4 ± 0.8 mV (R^2^ = 0.9985) for Na^+^, and 58.2 ± 0.9 mV (R^2^ = 0.9968) for K^+^ ions for three successful measurement, which are very close to the theoretically calculated slope value of the Nernst equation (i.e., 59.2) at 298 K for monovalent cations (Figs. 3D − F).

To assess the effectiveness of the Na_0.44_MnO_2_, and K_2_Co[Fe(CN)_6_] ionophores, the control experiments were performed with a membrane without corresponding ionophores. The response of two ionophore-free electrodes displays sensitivities of 39.8 ± 1.1 mV for Na^+^, and 46.1 ± 1.3 mV for K^+^ ions, which indicates a significant effect of the proposed ionophores in the fabrication of membrane electrodes. According to Table S3, the amount of the PANI as an ionophore was studied in H^+^-selective membrane composition. The obtained slope values show that the slope increases by increasing the PANI amount, and closer of slope to the Nernst value. These results indicated the significant effect of the PANI as an ionophore in the fabrication of H^+^- selective membrane.

According to the complex tissue of human sweat, the presence of different electrolytes may cause interference with accurate pH, Na^+^, and K^+^ detection. Therefore, investigation of the selectivity of proposed electrodes is essential for the multiplexed real-time monitoring of human sweat. The selectivity of three modified ISEs was checked in two different methods. In the first method, the potential responses of three modified ISEs were studied in a solution containing the constant concentration of H^+^, Na^+^, and K^+^ ions with different interference agents. In the second method, the selectivity coefficient of three modified ISEs was investigated in a solution containing the constant level of interference agent with different concentrations of H^+^, Na^+^, and K^+^ ions. The physiological level of common ions in sweat such as Na^+^, K^+^, Ca^2+^, NH_4_^+^, and Mg^2+^, as interfering ions for proposed sensors evaluation, is 20–100 mM, 4–24 mM, 0.5–3 mM, 0.5–8 mM, and 0.04–0.7 mM, respectively^19^. In the first method, interfering agents such as Na^+^ (100 mM), K^+^ (100 mM), Ca^2+^ (50 mM), NH_4_^+^ (100 mM), and Mg^2+^ (50 mM), were selected to study the selectivity of the pH, Na^+^, and K^+^ sensors in a solution containing 1.5 mM Na^+^ ion, 1 mM K^+^ ion, and the PBS with pH = 6.0, which is more than the physiological level of reported interference ions in sweat. Then, the potential responses of the pH sensor are evaluated after continuously adding the possible interfering ions into the PBS with pH = 6.0.

As shown in Fig. 3G, the potential responses demonstrate nearly no change. Also, the selectivity of the Na^+^ and K^+^ sensors was studied in the presence of above-mentioned interfering agents in the solution containing 2.5 mM Na^+^ and 1.5 mM K^+^, respectively (Fig. 3H and I). The obtained results exhibited a negligible effect of interference on the potential responses.

The fixed interference method was used to calculate selectivity coefficients, which are based on the potential measurement of solutions with a constant level of interference, a_B_, and varying activity of the primary ion, a_A_^38,39^. The obtained potential values are plotted against the activity of the primary ion. The intersection of the extrapolation of the linear portions of this curve will indicate the value of a_A_ which is to be used to calculate the Eq. (1):

1 $${\text{K}}_{{{\text{A}},{\text{B}}}}^{{{\text{Pot}}}} = \frac{{{\text{a}}_{{\text{A}}} }}{{\left( {{\text{a}}_{{\text{B}}} } \right)^{{\frac{{{\text{z}}_{{\text{A}}} }}{{{\text{z}}_{{\text{B}}} }}}} }}$$

where ${\text{K}}_{{{\text{A}},{\text{B}}}}^{{{\text{Pot}}}}$ is the potentiometric selectivity coefficient for the primary ion A against the interfering ion, B; z_A_ and z_B_ are charge numbers and *a*_A_ and *a*_B_ are the activities of the primary ion, A, and of the interfering ion, B. Table S4 shows the calculated selectivity coefficients of the pH, Na^+^, and K^+^ sensors in the presence of interfering ions, respectively. All the obtained selectivity coefficients for the pH sensor are in the range of 10^–4^ and, for Na^+^ and K^+^ sensors are in the range of 10^–3^, which indicates proposed sensors had a good selectivity and could be utilized for sweat analysis. The required selectivity coefficient ($\left( {{\text{K}}_{{{\text{A}},{\text{B}}}}^{{{\text{Pot}}}} } \right)_{{{\text{required}}}}$) related to pH, Na^+^, and K^+^ sensors was investigated^40^. The $\left( {{\text{K}}_{{{\text{A}},{\text{B}}}}^{{{\text{Pot}}}} } \right)_{{{\text{required}}}}$ is calculated based on the conventional Nimlsky-Eisenman equation, assuming an allowable error of 1% (0.01) in the worst case a maximum quantity of interfering ions, and a minimum quantity of primary ions within the physiological range by the following Eq. (2):

2 $$\left( {{\text{K}}_{{{\text{A}},{\text{B}}}}^{{{\text{Pot}}}} } \right)_{{{\text{required}}}} \le 0.01 \times \frac{{{\text{a}}_{{{\text{A}},{\text{min}}}} }}{{\left( {{\text{a}}_{{{\text{B}},{\text{max}}}} } \right)^{{\frac{{{\text{z}}_{{\text{A}}} }}{{{\text{z}}_{{\text{B}}} }}}} }}$$

where $\left( {{\text{K}}_{{{\text{A}},{\text{B}}}}^{{{\text{Pot}}}} } \right)_{{{\text{required}}}}$ is the required selectivity coefficients for the primary ion A against the interfering ion, B; z_A_ and z_B_ are charge numbers and *a*_A_ and *a*_B_ are the activities of the primary ion, A, and of the interfering ion, B. According to the physiological level of the ions mentioned above, the required selectivity coefficient was calculated. Table S4 shows the calculated required selectivity coefficients of the pH, Na^+^, and K^+^ sensors, respectively. The calculated required selectivity coefficients for most ions in the presence of interfering ions by the sensors are smaller than the selectivity coefficients. These results show that the proposed potentiometric sensors are capable of sweat analysis.

As shown in Figure S8, the long-term analysis stability of the pH, Na^+^, and K^+^ sensors was evaluated in the PBS (pH = 6.0), 10 mM Na^+^ solution, and 10 mM K^+^ solution for continuous operation over 5 h. The potentiometric responses of the pH, Na^+^, and K^+^ sensors showed high stability with only a little potential drift of about 0.2–1 mV/h. These results indicated that the pH, Na^+^, and K^+^ sensors are feasible for continually and accurately monitoring the pH, Na^+^, and K^+^ levels over 5 h.

Also, the formation of a water layer between the electrode substrate and the ion-sensing membrane significantly affects the electrode potential stability. The ISEs were conditioned for 24 h in 1 mM solution of the primary ion and then the electrode was sequentially exposed to 1 mM solutions of the primary ion for 60 min, the interfering ion for 180 min, and back to the primary ion for another 60 min^41^. All measurements were carried out in a stationary solution. For example, the potential drifts were recorded upon changing from the Na^+^ solution (1 mM) to an interfering ion solution (CaCl_2_; 0.1 M) then back again to the Na^+^ solution (1 mM). Results of the potentiometric aqueous layer test were shown in Figure S9A. The results show the potential stability of the Na^+^-selective membrane. This potential stability reflects the hydrophobic property of the Na^+^-selective membrane which eliminates the formation of an aqueous layer between the membrane interface with the electrode substrate. The same procedure was studied to investigate the water layer formed between the K^+^-selective membrane and pH-sensing membrane with electrode substrate in the presence of the corresponding membrane with K^+^ solution (1 mM) and PBS (pH = 5.0), respectively (Figures S9B and C). The interfering ions concentration was selected according to the selectivity coefficients for corresponding ions, and the initial pH value of PBS was chosen based on the level of pH in sweat (4.0–7.5)^42^. The initial jump of potential when changing solutions is related to the change in phase boundary potential at the membrane/solution interface and is a function of the selectivity coefficient and the concentrations of the primary and interfering ions^43^. A slightly positive potential drift was observed for ISEs when the primary ion solution was changed to the solution of the interfering ions. This phenomenon is due to the leaching of primary ions from the respective ion-selective membranes contaminating the highly discriminated interfering ion solution^44^.

The water contact angle of the Na^+^-selective membrane, K^+^-selective membrane, and H^+^-selective membrane were investigated to study their hydrophobic properties. The modified ISEs with the corresponding selected membranes were soaked for 0, 100, and 200 min and the water contact angle were measured by drop-casting a water drop on their surface (Figure S10). According to the literature, solids with a contact angle below 90°, are wetted with water and are classified as hydrophilic, and solids with a contact angle over 90° are considered hydrophobic^45^. Therefore, the proposed ion-selective membranes have acceptable hydrophobic properties.

In addition, the repeatability and the batch-to-batch reproducibility of pH Na^+^, and K^+^ sensors were studied (Figure S11). The pH, Na^+^, and K^+^ sensors demonstrated excellent repeatability with an average sensitivity of 55.2, 63.4, and 58.2 mV/decade, with standard deviation (SD) values of 0.5, 0.8, and 0.7 mV, respectively (Figures S11A-C). Also, the E^0^ and the slope of the calibration curve were compared for three parallel batches of modified ISEs for the study of reproducibility. The batch-to-batch reproducibility of 9 electrodes (three batches) for each modified ISE was shown in Figures S11D-F. Three different batches of Na^+^, K^+^, and pH sensors were prepared weekly to test. Comparing the potentiometric responses of three batches shows practically the Nernstian response for all 9 electrodes (Table S5). Despite strictly following the same fabrication protocols, most often significant changes in the E^0^ values are observed for the different electrode batches. Three batches of pH sensor (n = 3) had excellent single-batch E^0^ reproducibility (SD = 0.5 − 0.9 mV), while the average E^0^ values of the three batches almost differed and resulted in a large interbatch SD of 1.3 mV. The same results were observed for the single-batch E^0^ reproducibility of Na^+^ and K^+^ sensors, in which the single-batch SD values were much lower than the interbatch SD. Note, the electrodes were immersed in 1 mM corresponding ions for 24 h prior to the calibration. The obtained results indicate that the proposed sensors have reliable preparation with acceptable batch-to-batch potential reproducibility.

The sensitivity of the sensors under different ionophore amounts was studied. For this goal, the potentiometric responses of sensors with different ionophore amounts were investigated in different corresponding ion concentrations (Figure S12). The obtained slopes were listed in Table S6. The sensitivity of Na^+^ and K^+^ sensors was slightly deteriorated by increasing the ionophore, which can relate to the resistance increase of the Na^+^ and K^+^ ion-selective membrane (Figures S12, A and B). Figure S12, C shows the sensitivity of the pH sensor. It shows that from 5 to 20 mg of PANI as an ionophore has negligible influence on pH sensing performance while having a remarkable influence in the 25 mg of PANI. Increasing sensitivity and super-Nernstian behavior are due to the increase in free ionophore concentration and the depletion of primary ions near the membrane.

The redox sensitivity measurements were carried out for the ISEs covered with H^+^-selective membrane, Na^+^-selective membrane, and K^+^-selective membrane. The potential of modified ISE with Na^+^-selective membrane was recorded in solutions containing fixed 0.01 M NaCl ionic strength, and a redox couple FeCl_3_. 6H_2_O and FeCl_2_. 4H_2_O at a total concentration of 1 mM with the log of Fe^3+^/Fe^2+^ ratio equal to -1, -0.5, 0, 0.5, and 1 (Figure S13). Also, the redox sensitivity of modified ISEs with K^+^-selective membrane, and H^+^-selective membrane were investigated by similar procedure with changing the 0.01 M KCl, and PBS (pH = 6.0) instead 0.01 M NaCl, respectively. As shown in Figure S13, no redox sensitivity was observed for the modified ISEs, and ISEs indicated a potential change of almost 1 mV/decade during the experiment. This result clearly shows that the redox system in the solution did not affect the potential of ISEs and there was no direct contact between the transducer layer and the sample solution.

The hysteresis of the potentiometric sensors is crucial for accurate analysis of concentrations of target analytes. The hysteresis analysis is conducted by exposing the sensors to various levels of corresponding target ions cyclically. The Na^+^ concentration varied between 1 and 130 mM, and the results demonstrated good reversibility with a negligible average RSD of 0.5% (Figure S14, A). Similarly, K-ISE (0.5 − 80 mM) and pH sensors (pH 4 − 7.5) displayed good hysteresis with average RSDs of 0.4% and 0.3%, respectively (Figure S14, B and C).

The ISE sensors due to their wearable applications may constantly experience bending; therefore, it is important to evaluate electrochemical sensing performances while undergoing deformation. The detection performance of the fabricated sensing for pH, Na^+^, and K^+^ detection was performed under bending states (Figure S15A). As seen in Figure S15B the pH sensor was tested in PBS with different pH from 4.0 to 8.0 under stretch. It shows that stretch has negligible influence on pH sensing performance and shows the Nernstian response (-55.4 ± 0.7,). Similar results were also observed for Na^+^ and K^+^ sensors, both could demonstrate Nernstian response with an average of 63.5 ± 0.9 and 58.4 ± 0.8 in the solution containing Na^+^ from 1 to 100 mm and K^+^ from 1 to 80 mm when stretched, respectively (Figures S15C, and D).

The shelf-life of the sensors were investigated after they were fabricated. Ideally, the sensors should be able to be stored at room temperature and have a long shelf-life. Therefore, the shelf-life of the potentiometric sensor was determined at room temperature. The modified ISEs with the corresponding selected membranes were soaked in 1 mM Na^+^ ion, 80 mM K^+^ ion, and the PBS with pH = 6.0 during storage. Then potentiometric response of the proposed ISEs were tested for Na^+^, K^+^, and pH at day 1, day 7, day 14, and day 21. Figure S16 shows the potentiometric response of the Na^+^, K^+^, and pH sensors at different days. ISEs displayed a potential change with RSDs less than 5% during the experiment. Clearly, the proposed ISEs were shelf-life to three weeks when stored at room temperature.

### Discrimination analysis

Among the different exploratory data analysis methods, the first choice is principal component analysis (PCA), as a linear, unsupervised pattern recognition, which decomposes a higher dimensional data matrix into the product of two orthogonal matrices of lower dimensions. It produces a set of unique variances (i.e., principal components, PCs) using a linear combination of the original variables^46–48^. PCA analysis was performed by using MATLAB R2018a (version 9.4). Figure S17 shows the three-dimensional PCA plot for discriminating potentiometric responses of the Na^+^, K^+^, and pH sensors in the 1, 15, and 80 mM of Na^+^ solutions, 1, 15, and 80 mM of K^+^ solutions, and PBS with three pH levels of 4.0, 60, and 7.5. Variance analysis revealed that 100% variances could be expressed by the first 3 PCs (PC1; 99.97%, PC2; 0.02%, PC3; 0.01%). Figure S17 shows that potentiometric responses of the pH, Na^+^, and K^+^ sensors are completely discriminated in the three-dimensional factor space. Therefore, it can be concluded that the H^+^, Na^+^, and K^+^ ions are distinctly separated based on the reversible transition of ions in the corresponding ionophore.

Furthermore, the correlation coefficients can be used as a parameter for the investigation of whether or not to is a significant relationship between the potentiometric responses of Na^+^, K^+^, and pH sensors. The Python programming language version 3.10.12 on the Colab platform was utilized. The Numpy and Scipy libraries were used to calculate the correlation coefficient, P-value and regression calculation, and the Matplotlib library was used to display the scatter plot. The correlation strength is determined by the closeness of “r” to 1 in absolute value. The correlation strength for the various values of “r” is as follows: (1) r > 0.9, very high correlation, (2) 0.7 < r < 0.9, high correlation, (3) 0.5 < r < 0.7, moderate correlation, (4) 0.3 < r < 0.5, low correlation, and (5) r < 0.3, negligible correlation^49^. Correlation plots of potentiometric responses of Na^+^, K^+^, and pH sensors for the 1, and 80 mM of Na^+^ solutions, 1, and 80 mM of K^+^ solutions, and PBS with two pH levels of 4.0, and 7.5 are shown in Figure S18. Table S7 shows the obtained correlation coefficient for the potentiometric responses of Na^+^, K^+^, and pH sensors. As shown in Table S7, the weak correlation coefficients (r < 0.3) and the P-value > 0.05 indicate the lack of a significant relationship between potentiometric responses of Na^+^, K^+^, and pH sensors. Therefore, the proposed potentiometric sensors can simultaneously determine Na^+^, K^+^, and H^+^ ions in sweat because of the weak correlation coefficient between their potentiometric responses.

### Sensing mechanism evaluation

The response mechanism of Na^+^, K^+^, and pH sensors relies on the change in the membrane potentials of Na^+^, K^+^, and H^+^, respectively, which results from changes in the corresponding ion levels. As shown in Figure S19. the nanostructured Na_0.44_MnO_2_ has an orthorhombic lattice structure. The tunnel type of Na_0.44_MnO_2_ is particularly attractive due to its unique large tunnels suitable for sodium intercalation. The unit cell of Na_0.44_MnO_2_ is made up of MnO_5_ square pyramids (containing Mn^3+^ ions) and MnO_6_ octahedra (containing Mn^4+^ ions and half of the Mn^3+^ ions), which formed two different kinds of tunnels, a large S-shaped tunnel, and a smaller pentagon-shaped tunnel. The, while the other sites (Na-3) located in the smaller pentagon tunnel are not mobile. According to this crystal structure, the main path for Na^+^ ions diffusion is along the c-direction^50^. Therefore, the mobility of Na^+^ ions in the Na-3 sites leads to a change in the potential value. The proposed mechanism can be written as Eq. (3): ^51^

3 $${\text{Na}}_{0.44} {\text{MnO}}_{2} { }\underset{{{\text{incorporating}}}}{\overset{{{\text{remove}}}}{\longleftrightarrow}}{\text{Na}}_{{\text{x}}}^{ + } + {\text{Na}}_{{0.44 - {\text{x}}}} {\text{MnO}}_{2} + {\text{ e}}^{ - }$$

K_2_Co[Fe(CN)_6_] can serve as a promising alternative for K^+^ ionophore owing to the abundant storage of K^+^ ions. The potentiometric response mechanism of K_2_Co[Fe(CN)_6_] toward K^+^ ions is the reversible inclusion of K^+^ ions in the K_2_Co[Fe(CN)_6_] lattice. Therefore, K_2_Co[Fe(CN)_6_] as a K^+^ ionophore has the potential to monitor the K^+^ ion's level of sweat in real-time. The proposed mechanism can be written as Eq. (4):

4 $${\text{K}}_{2} {\text{CoFe}}^{{{\text{II}}}} \left( {{\text{CN}}} \right)_{6} \underset{{{\text{incorporating}}}}{\overset{{{\text{remove}}}}{\longleftrightarrow}}{\text{KCoFe}}^{{{\text{III}}}} \left( {{\text{CN}}} \right)_{6} + {\text{e}}^{ - } + {\text{K}}^{ + }$$

The employment of a PANI as an active material for selective pH measurement is based on the proton sensitivity of PANI, which is a reversible process. PANI has six different forms including leucoemeraldine base (LEB, totally reduced), leucoemeraldine salt (LES), pernigraniline base (PAB), pernigraniline salt (PAS, totally oxidized), emeraldine base (EB, intermediate form), and emeraldine salt (ES, conductive and protonated form). Among these forms, the EB form is easily protonated with protons to the ES form because of the presence of imine and amine functional groups on the backbone of PANI. The pH response mechanism of polyaniline-based sensors depends on the reversible transition from the emeraldine salt to the emeraldine base. Therefore, PANI has the potential to monitor the pH value of sweat in real time. The mechanism can be written as Eq. (5):^52^

5 $${\text{PANI}}_{{\left( {{\text{EB}}} \right)}} + {\gamma H}^{ + } + {\text{ne}}^{{ - { }}} { }\underset{{{\text{incorporating}}}}{\overset{{{\text{remove}}}}{\longleftrightarrow}}\left[ {{\text{PANI}}.{\text{H}}} \right]_{{\left( {{\text{ES}}} \right)}}^{{\left( {{\upgamma } - {\text{n}}} \right)}}$$

### Out-body sweat test

**Figure 4:**
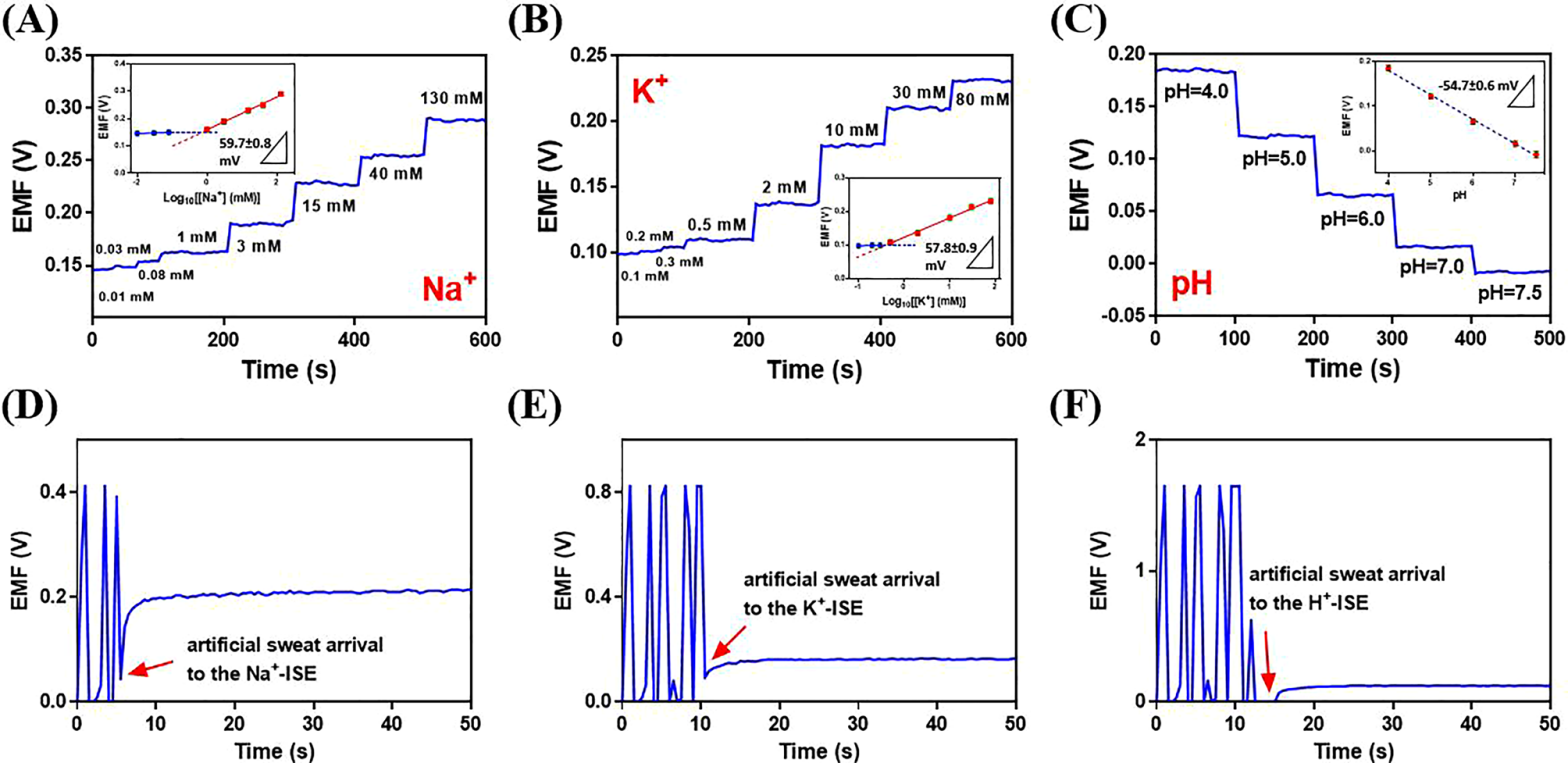
Integrated test results of the fabricated wearable potentiometric sensor for analysis of (**A**) Na^+^, (**B**) K^+^, and (**C**) pH in NaCl, KCl, and PBS solutions. The insets in (**A**) − (**C**) show the corresponding calibration plots of the sensors. Integrated test results ofthe fabricated wearable potentiometric sensor for the detection of (**D**) Na^+^, (**E**) K^+^, and (**F**) pH in artificial sweat.

The pH, Na^+^, and K^+^ sensors were utilized for the out-body detection of pH, Na^+^, and K^+^ levels when the sensing ability of the fabricated proposed nanomaterials was confirmed. The analysis of out-body sweat was performed by using a corresponding standard solution with various concentrations of Na^+^, and K^+^ and various pHs to demonstrate the possibility of real human sweat analysis (Fig. 4A–C). The obtained data using the integrated PCB can be collected and transferred to a smartphone wirelessly. These results display that the sensors demonstrated a linear potentiometric response, and their sensitivity in the artificial sweat is much similar to the aqueous solution.

As shown in Fig. 4A–C, the output potentiometric signals of three sensors integrated into the platform displayed excellent linear response with the logarithm of the Na^+^ and K^+^ concentrations and pH, with sensitivities of 59.7 ± 0.8 mV (R^2^ = 0.9953) for Na^+^, and 57.8 ± 0.9 mV (R^2^ = 0.9939) for K^+^ ions, and 54.7 ± 0.6 mV (R^2^ = 0.9970) for H^+^ ions. In the study of integrated platform performance, the potentiometric responses of the pH sensor in PBS (0.1 M) with pH ranging from 4 to 7.5, the Na^+^ sensor in 1 − 130 mM NaCl solution, and the K^+^ sensor in 0.5 − 80 mM KCl solution, were investigated. Therefore, according to the literature, the level of pH in sweat during sport monitoring is 4.0–7.5, and for Na^+^, and K^+^ is 20–100 and 3–10 mM^42^, respectively the as-prepared potentiometric sensor can detect the concentration of them in the sweat. The detection limit of the proposed potentiometric sensor for Na^+^ and K^+^ was calculated at 0.57 and 0.39 mM, respectively, which is lower than the physiological level of Na^+^ and K^+^ in sweat. These results indicated that the three sensors integrated into the platform have all provided excellent responses during the simultaneous detection of analytes. In addition, the proposed sensor displayed a short response time of < 8 s for three analytes, which is excellent for utilizing in on-body sweat tests. A brief comparison between the proposed potentiometric sensor with similar potentiometric devices, which were reported in previous studies is presented in Table S8. The proposed sensor design can measure H^+^, Na^+^, and K^+^ in sweat, indicating that the performance of the used ionophores is comparable to traditional ionophores reported for these ions. According to Table S7, the proposed sensors show an expanded linear range and better sensitivity compared to some reported sensors. Furthermore, the sweat sample can move and arrive at the electrodes by using a paper strip via the capillary effect. This design prevents the direct attachment of the surface of modified electrodes to the skin. Also, thin paper sandwiched between two PVC sheets can hinder the evaporation of sweat and solve the production problem of sweat at different intervals of time. Then, the performance of the integrated potentiometric sensor for multiplexed monitoring of pH, Na^+^, and K^+^ levels was evaluated by artificial sweat (Fig. 4D–F). Artificial sweat was prepared according to the description of the experimental section. The pH, Na^+^, and K^+^ levels were calculated using the corresponding calibration plots. The potentiometric responses were noisy before the arrival of artificial sweat, and then a stable response was observed. The levels of Na^+^, K^+^, and pH were calculated at 9.8 mM, 2.9 mM, and 5.5, respectively. The artificial sweat was analyzed using the AAS method and pH meter for validation of the obtained result. The obtained values of the integrated potentiometric sensor for Na^+^, K^+^, and pH were in agreement with the obtained results of AAS for Na^+^ (10.1 mM), K^+^ (3.0 mM), and pH meter for pH (5.6) in the initial artificial sweat. Therefore, the complex matrix of artificial sweat had little effect on the multiplex monitoring of analytes by the proposed potentiometric sensor. Furthermore, these results clearly show that the markers in artificial sweat have no side interaction such as the adsorption process with the sandwiched thin paper in the platform.

### On-body sweat test

**Figure 5:**
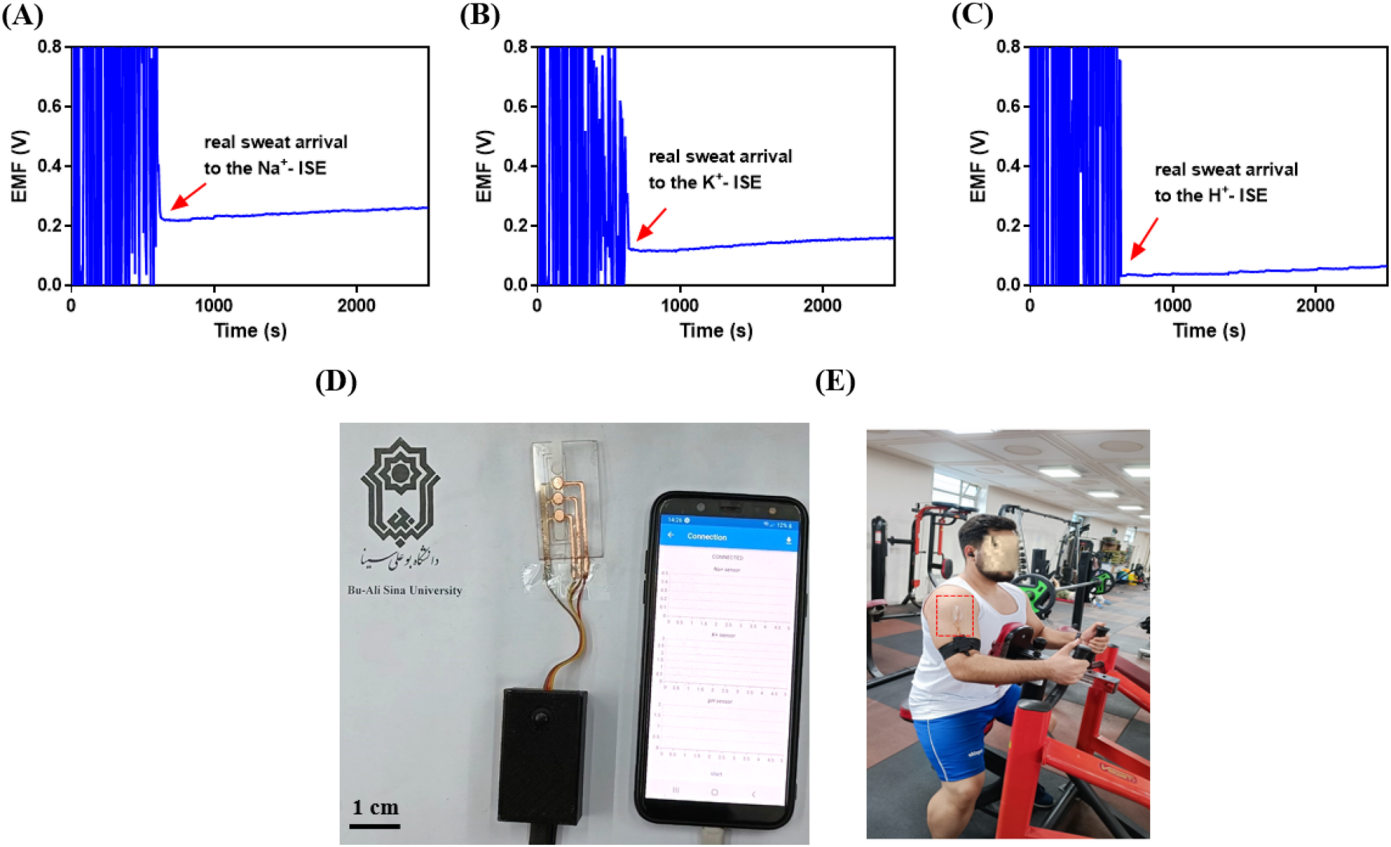
Integrated test results of the fabricated wearable potentiometric sensor for analysis of (**A**) Na^+^, (**B**) K^+^, and (**C**) pH in real sweat samples. (**D**) Photograph of the potentiometric sensor and host smartphone. and (**E**) On-body monitoring of Na^+^, K^+^, and pH in real sweat sample by the proposed potentiometric sensor.

As shown in Fig. 5A–C, the on-body sweat analysis by a proposed potentiometric sensor was performed on the on arm of the volunteer with informed consent to participate in 40 min of exercise. The produced sweat at different intervals of time was collected by a thin layer of paper into the platform and arrived at the electrodes based on capillary effect. Real-time potentiometric responses were wirelessly transmitted to a host smartphone (Fig. 5D). The potentiometric responses were noisy before the onset of sweating, and then a stable response was observed. The potential of electrodes was changed after the starting of sweat during the exercise for 40 min which indicates the changing of pH and Na^+^ and K^+^ levels (Fig. 5E). The surface of the electrodes reaches a saturated state over time during sports activity and causes the potential changes to be less and almost constant. The pH, Na^+^, and K^+^ levels were calculated using the corresponding calibration plots saved in the software memory. The levels of Na^+^, K^+^, and pH were calculated at 58.9 mM, 7.8 mM, and 6.1, respectively. Figure S20 presents the potentiometric response of the proposed potentiometric sensor towards Na^+^, K^+^, and pH for volunteer 2. The levels of Na^+^, K^+^, and pH were calculated at 69.8 mM, 6.3 mM, and 5.8, respectively. The obtained results of real sweat analysis for Na^+^, K^+^, and pH levels by the proposed potentiometric sensor were in the range of the healthy individual body. Therefore, these observations approve the attractiveness of the developed wearable potentiometric sensor for real-time monitoring of Na^+^, K^+^, and pH in sweat samples.

## Conclusions

This study developed an integrated potentiometric sensing platform for non-invasive multiplexed detection of pH, Na^+^, and K^+^ in human sweat. The proposed potentiometric sensor is capable of real-time Na^+^, K^+^, and pH monitoring with high sensitivity (59.7 ± 0.8 mV/decade for Na^+^, 57.8 ± 0.9 mV/decade for K^+^, and 54.7 ± 0.6 mV/pH for H^+^), excellent stability (5 h), and good reproducibility by using Na_0.44_MnO_2_, K_2_Co[Fe(CN)_6_], and PANI as an ionophore, respectively. The XRD, FESEM, and TEM techniques confirmed the successful synthesis of ionophores. The electrode patterns were printed on the thin flexible PVC sheet. Thin paper sandwiched between two PVC sheets can enhance sweat sampling via the capillary effect of thin paper without evaporating sweat and solve the production problem of sweat at different intervals of time. This wearable sensor is integrable with a PCB, which enables in situ signal collection and transmission in real time to the smartphone through a Wi-Fi access point. The wearable potentiometric sensor in both out-body and on-body tests indicated reliable sweat assessment. The sweat analysis conducted on-body indicated that the proposed ionophores can use for multiplexed monitoring of Na^+^, K^+^, and pH. Miniaturization of the sensor bed and also the use of a flexible supercapacitor instead of a battery are important for future projections.

## Supplementary file

Supplementary material (the vendor information of chemicals and apparatus; The methods used for the synthesis of PANI, Na_0.44_MnO_2_, and K_2_Co[Fe(CN)_6_]; The optical images of the electrodes array (i.e. working electrodes, and quasi-reference electrode), and spacers; The above view of the accessory box of the potentiometric sensor; The FESEM images of different layers of Ag/AgCl/PVB; The FESEM images of Ag NPs layers, and Cu NPs layers on the PVC sheet; The cross-sectional images of step by step fabrication of Ag/AgCl/PVB quasi-reference electrode; Potentiometric responses of Ag/AgCl/PVB electrode and CV of 5 mM [Fe(CN)_6_]^3−/4−^ vs. Ag/AgCl/PVB quasi-reference electrode in 0.1 M KCl solution on bare GCE at a scan rate of 0.1 V s−1; Long-term stability of the wireless device for H^+^, Na^+^ and K^+^ detection for 5 h; Aqueous layer test of the potentiometric sensors; The water contact angle measurements on the ion selective membranes; The repeatability and batch-to-batch reproducibility of pH-sensor, Na^+^-sensor, and K^+^ sensor; The sensitivity of sensors under different ionophore amounts; The redox sensitivity test of sensors; The hysteresis test of sensors; Representation of the structure of Na_0.44_MnO_2_; On-body sweat test for volunteer 2; Membrane composition optimization of pH-sensor, Na+-sensor, and K^+^ sensor; The estimated selectivity coefficients for the pH, Na^+^ and K^+^ sensors; Comparison of the performances of the wearable potentiometric sensors with this work) is available in the online version of this article.

### Supplementary Information

Supplementary Information 1.

Supplementary Video 1.

## Data Availability

Data is provided within the manuscript or supplementary information files.
