# Supplementary material for: Wireless wearable potentiometric sensor for simultaneous determination of pH, sodium and potassium in human sweat
Source: Sci Rep. 2024 May 21;14:11526. doi: 10.1038/s41598-024-62236-3 (PMC11109153; doi:10.1038/s41598-024-62236-3)
Supplement: Supplementary file 1 — Supplementary Information 1. [file 41598_2024_62236_MOESM1_ESM.doc]

# Electronic Supplementary Material

| **Wireless Wearable Potentiometric Sensor for Simultaneous Determination of pH, Sodium and Potassium in Human Sweat** |
| --- |
| Nahid Rezvani Jalal^a^, Tayyebeh Madrakian^*a^, Mazaher Ahmadi^*a^, Abbas Afkhami^a^, Sina Khalili^a^, Morteza Bahrami^b^ and Majid Roshanaei^c^  ^a^Faculty of Chemistry, Bu-Ali Sina University, Hamedan 6517838695, Iran  ^b^Department of Computer Engineering, Faculty of Engineering, Bu-Ali Sina University, Hamedan 6517838695, Iran  ^c^Biomedical Engineering Department, School of Electrical Engineering, Iran University of Science and Technology, Tehran 1684613114, Iran |

*Corresponding author: E-mail: [madrakian@basu.ac.ir](mailto:madrakian@basu.ac.ir), m.ahmadi@basu.ac.ir

**Chemicals and Apparatus**

Aniline monomer with a purity of 100%, Ammonium persulfate ((NH_4_)_2_S_2_O_8_) with a purity ≥ 98%, Hydrochloric acid (HCl) with 37% purity, Cobalt(II) acetate tetrahydrate (Co(OAc)_2_. 4H_2_O) with a purity ≥ 98%, Potassium ferricyanide (K_3_[Fe(CN)_6_) with 99% purity, trisodium citrate dihydrate (C_6_H_5_Na_3_O_7_. 2H_2_O) with a purity ≥ 99%, Potassium chloride (KCl) with a purity of 99.99%, ammonium chloride (NH_4_Cl) (purity ≥ 99.5%), Magnesium nitrate hexahydrate (Mg(NO_3_)_2_. 6H_2_O) with 99% purity, Iron(II) chloride tetrahydrate (FeCl_2_. 4H_2_O) with 98% purity, Iron(III) chloride hexahydrate (FeCl_3_. 6H_2_O) with a purity ≥ 99%, Polyvinylpyrrolidone K-30 (PVP) with analytically pure, sodium chloride (NaCl) with a purity ≥ 99%, calcium chloride (CaCl_2_) with a purity ≥ 97%, Sodium carbonate (Na_2_CO_3_) with a purity ≥ 99%, Sodium bicarbonate (NaHCO_3_) with 99% purity, tetrahydrofuran (THF) with a purity of 99.9%, dibutyl phthalate (DBP) with 99% purity, oleic acid (OA) with a purity ≥ 99%, nitro benzene (NB) with 99% purity, dioctyl phthalate (DOP) (> 99% purity), disodium hydrogen phosphate (Na_2_HPO_4_) with a purity ≥ 99%, polyvinyl chloride (PVC) with analytically pure, sodium tetraphenyl borate (NaTPB) with a purity ≥ 99.5%, and manganese carbonate (MnCO_3_) with a purity ≥ 99.9% were purchased from Merck Company (Darmstadt, Germany). Urea, glucose, lactic acid, and uric acid were purchased from Sigma–Aldrich Company (St. Louis, MO, USA). Acetone was purchased from Mojallali Company (Tehran, Iran). Polyvinyl butyral (PVB) with analytically pure was purchased from Sigma-Aldrich (St. Louis, MO, USA). Also, silver ink was purchased from SKC Company (Seoul, Korea). All other chemicals and reagents utilized were of analytical grade. Deionized water was utilized in all experiments.

Chronopotentiometric studies were carried out by using a potentiostat/galvanostat Autolab (PGSTAT 302 N model, Netherlands) that was controlled by NOVA 1.11 software. The electromotive force (EMF) of the potentiometric sensor was measured by utilizing a digital multimeter. To investigate the chemical structures, a Perkin Elmer (Model Spectrum GX) infrared spectrometer was used to perform Fourier transform infrared (FT-IR) analysis in the 4000-400 cm-1 range. XRD diffractometer (GNR, APD 2000 PRO, Italy) with Cu Kα radiation was used to collect powder X-ray diffraction (XRD) patterns. Field emission scanning electron microscopy (FESEM) images and energy dispersive X-ray (EDX) studies to examine the surface morphologies and elemental analysis were recorded at 30 keV by using a TESCAN MIRA3 LMU instrument (Czech Republic). The atomic absorption spectroscopy (AAS, Agilent Technology 200 Series AA, USA) was utilized to examine the determination of Na^+^ and K^+^ ions in the artiﬁcial and real sweat. Also, a Metrohm 713 pH meter was employed to adjust the pH of the solutions.

**Synthesis of PANI**

The PANI was prepared to the method reported in the literature with some modifications ^1^. PANI was synthesized using two different solutions, A and B. At first, 0.5 mL of aniline monomer was combined homogeneously with 50 mL of HCl (1 M) to get solution A. Solution B was prepared by combining 1.55 g of (NH4)_2_S_2_O_8_ and 50 mL of HCl (1 M). In the ice bath, then solution B was added to solution A while the stirring was maintained for 8 h. The polymerization process ended up with a green precipitate of PANI. Finally, the green precipitate of PANI was filtered and rinsed 3 times with deionized water and acetone, respectively. Then, the obtained product was dried in a vacuum oven at 60 ˚C for 30 minutes.

**Synthesis of Na_0.44_MnO_2_**

A simple solid-state reaction technique was used to synthesize Na_0.44_MnO_2_ ^2^. First, 1.18 g of MnCO_3_ and 0.24 g of Na_2_CO_3_ were combined and annealed at 300 °C for 8 h, followed by 800 °C for 9 h. The black powder was obtained after cooling to room temperature.

**Synthesis of** **K_2_Co[Fe(CN)_6_]**

The K_2_Co[Fe(CN)_6_] was prepared to the method reported in the literature ^3^. Briefly, 0.3 g of Co(OAc)_2_. 4H_2_O, 0.37 g of C_6_H_5_Na_3_O_7_.2H_2_O, and 3.0 g PVP were dissolved in 40 mL of deionized water and stirred for 10 minutes (Solution A). After that, 0.2 g of K_3_[Fe(CN)_6_] was dissolved in 60 mL deionized water and stirred for 10 min (Solution B). Next, Solution B was added to Solution A under magnetic stirring for 15 s at room temperature and mixed for 1 min. Then, the resulting solution was mixed and aged at 80 °C for 24 h. In the last step, the obtained products were filtered and rinsed several times with deionized water and ethanol before being dried overnight in a vacuum oven at 70 °C.


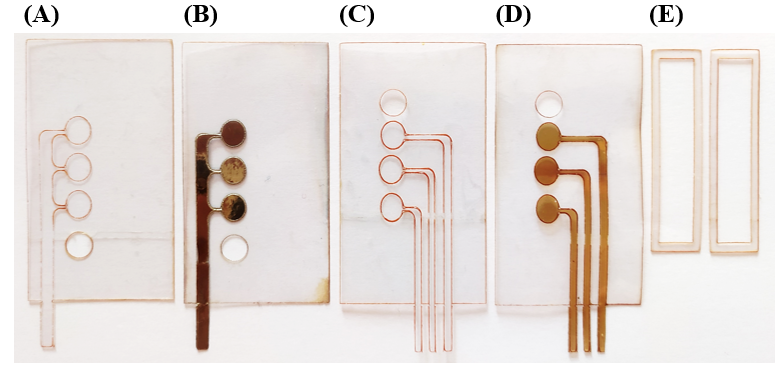


**Figure S1.** The optical images of A) the pattern of the quasi-reference electrode, B) the coated quasi-reference electrode with Ag NPs layers, C) the pattern of working electrodes, D) the coated of working electrodes with Cu NPs layers on the PVC sheet, and E) spacers.


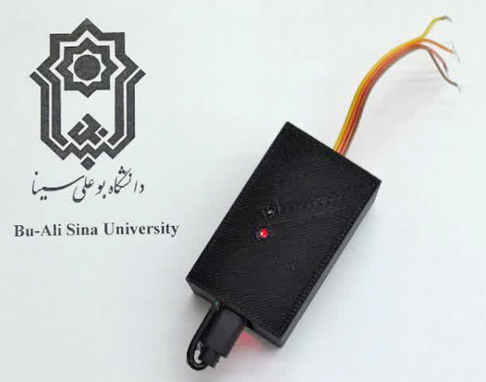


**Figure S2.** The above view of the accessory box of the potentiometric sensor.


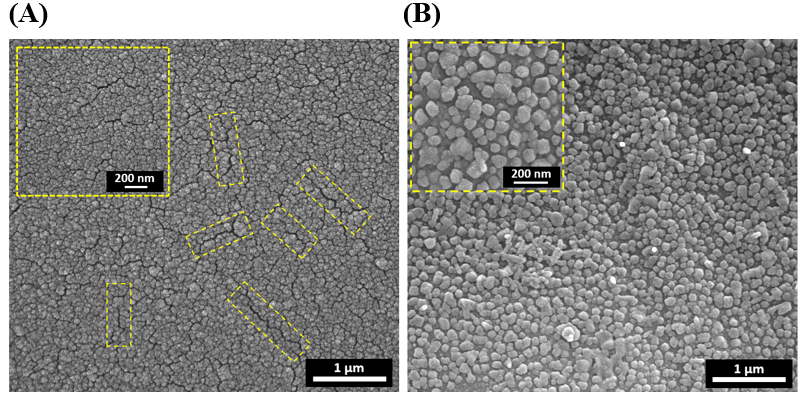


**Figure S3.** The FESEM images of A) Ag NPs layers and B) Cu NPs layers on the PVC sheet.


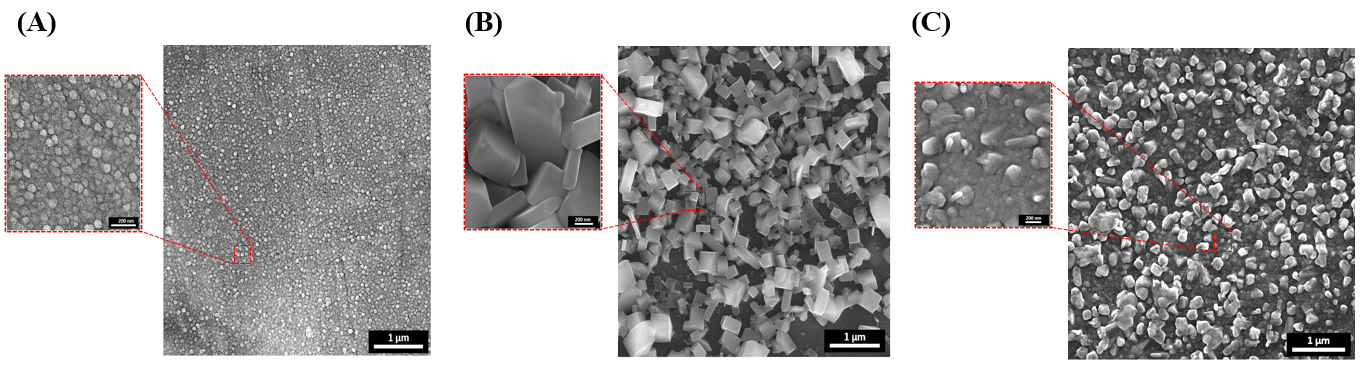


**Figure S4.** FESEM images of (A) Ag NPs layers, (B) Ag/AgCl layers, and (C) Ag/AgCl/PVB layers; (the insets show the high magnification).


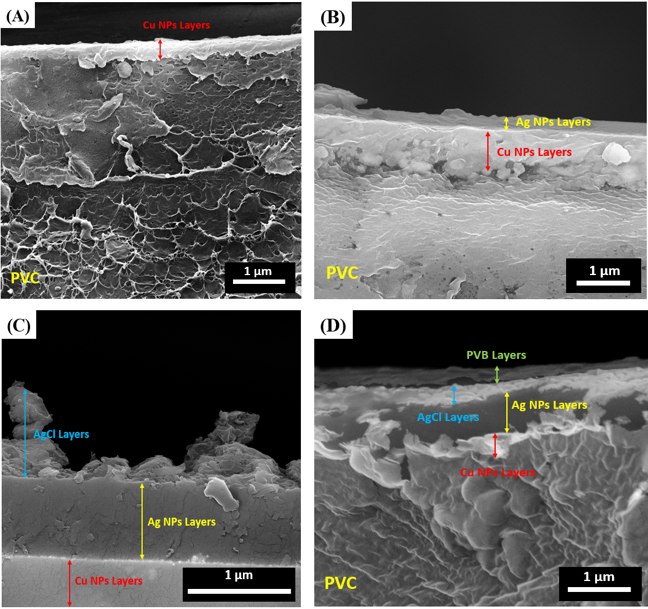


**Figure S5.** The cross-sectional images of step-by-step fabrication of Ag/AgCl/PVB quasi-reference electrode, A) Cu NPs/PVC, B) Ag NPs/Cu NPs/PVC, C) AgCl/Ag NPs/Cu NPs/PVC, and D) PVB/AgCl/Ag NPs/Cu NPs/PVC, denoted as Ag/AgCl/PVB.


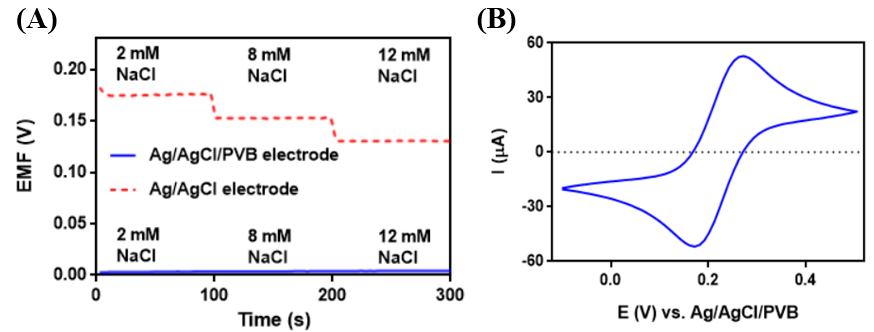


**Figure S6.** (A) Potentiometric responses of Ag/AgCl/PVB electrode and Ag/AgCl electrode in diﬀerent NaCl solutions; (B) CV of 5 mM [Fe(CN)_6_]^3−/4−^ vs. Ag/AgCl/PVB quasi-reference electrode in 0.1 M KCl solution on bare GCE at a scan rate of 0.1 V s^−1^.


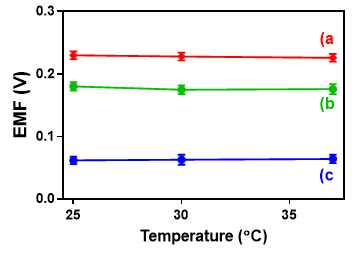


**Figure S7.** The potentiometric responses of A) Na^+^-sensor, B), K^+^-sensor and C) pH-sensor under different temperatures.


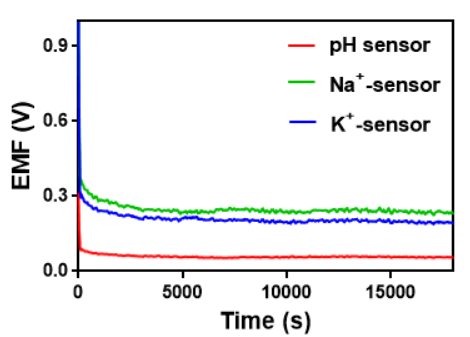


**Figure S8.** Long-term stability of pH, Na^+^, and K^+^ sensors for 5 h.


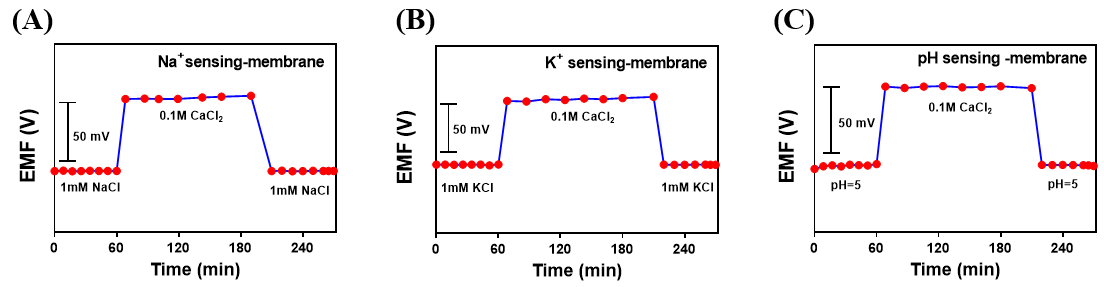


**Figure S9.** Aqueous layer test of potentiometric sensor in A) Na^+^ ion solution (1 mM), B) K^+^ ion solution (1 mM), C) PBS (pH=5.0), and interfering ion (Ca^2+^) solution (Scales characterized for plots are defined based on their Y-axis).


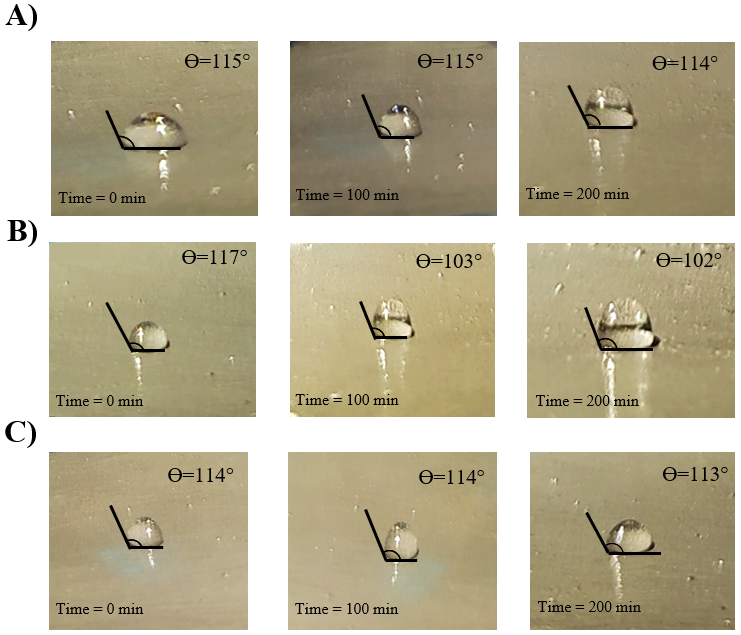


**Figure S10.** The water contact angle measurements on A) Na^+^- selective membrane, B), K^+^- selective membrane, and C) H^+^-selective membrane.


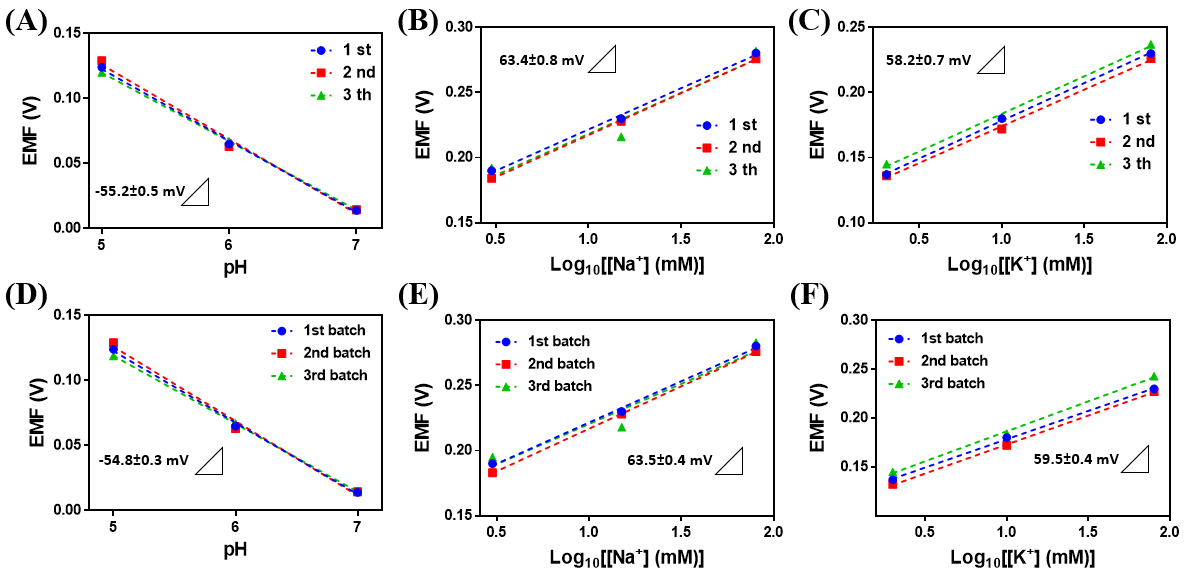


**Figure S11.** The repeatability of A) pH-sensor, B) Na^+^-sensor, and C) K^+^ sensor (n = 3). Batch-to-batch reproducibility for three fabrication batches of D) pH-sensor, E) Na^+^-sensor, and F) K^+^ sensor (n = 3 electrodes).


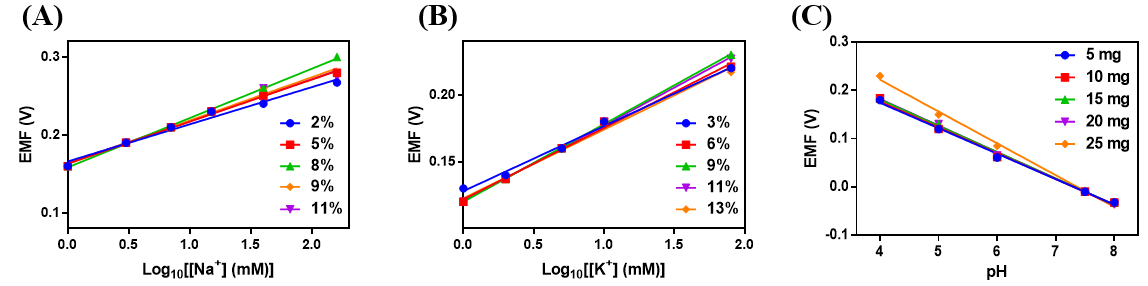


**Figure S12.** The sensitivity of A) Na^+^-sensor, B), K^+^-sensor and C) pH-sensor under different ionophore amounts.


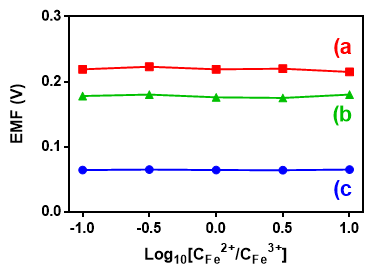


**Figure S13.** The redox sensitivity test of a) Na^+^-sensor, b), K^+^-sensor, and c) pH-sensor in a solution containing 1 mM total concentration of Fe^2+^/Fe^3+^ redox couple, and constant ionic background of 0.01 M NaCl, 0.01 M KCl, and PBS (pH=6.0), respectively.


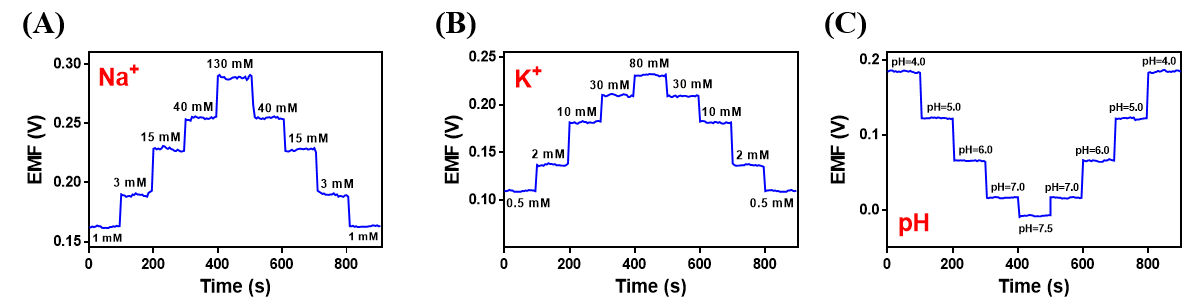


**Figure S14.** The hysteresis test of a) Na^+^-sensor, b), K^+^-sensor, and c) pH-sensor in changing the concentration of target ions cyclically, respectively.


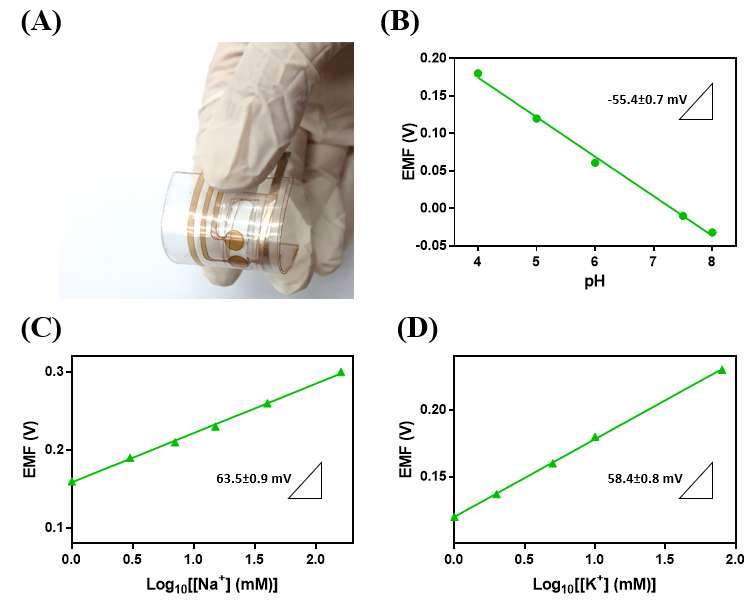


**Figure S15.** The potentiometric responses of A) Na^+^-sensor, B), K^+^-sensor and C) pH-sensor under deformation.


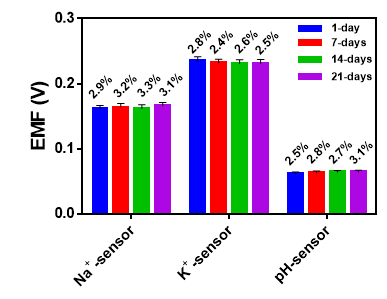


**Figure S16.** The shelf-life of A) Na^+^-sensor, B), K^+^-sensor and C) pH-sensor in different days.


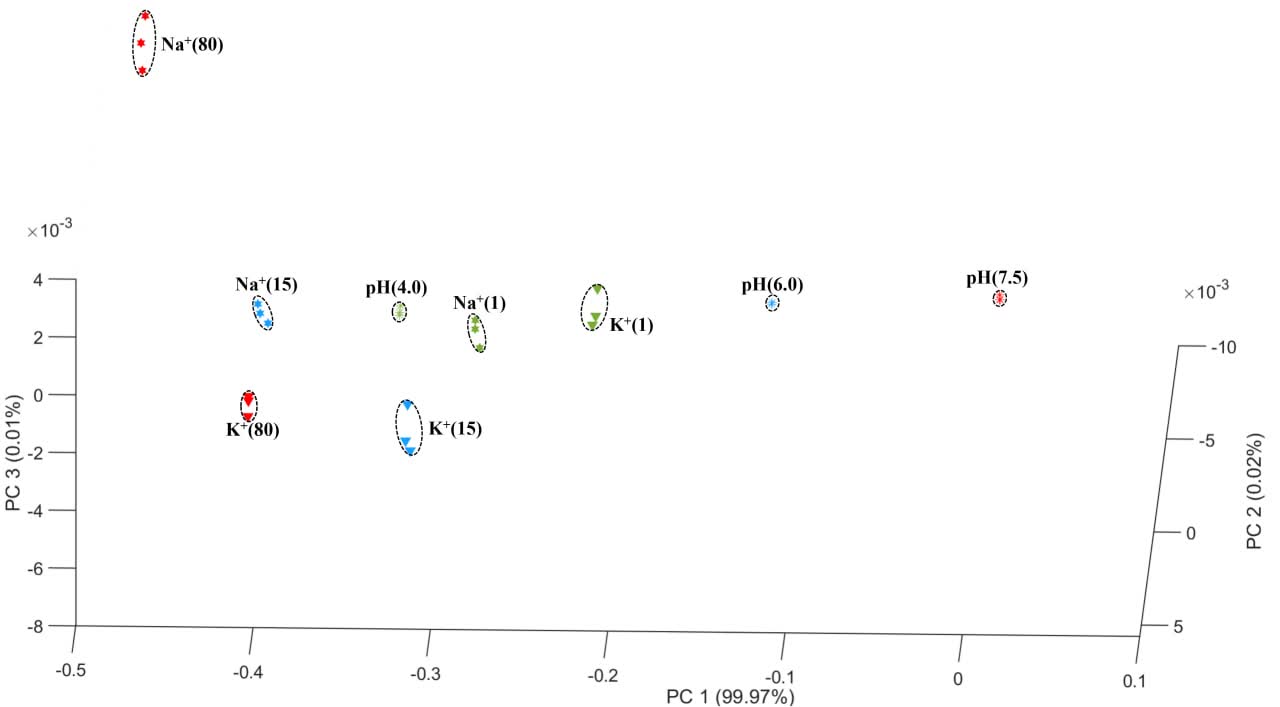


**Figure S17.** Clustering of potentiometric responses of the pH, Na^+^, and K^+^ sensors in the 3D PCA score plot in the 1, 15, and 80 mM of Na^+^ solutions, 1, 15, and 80 mM of K^+^ solutions, and PBS with three pH levels of 4.0, 60, and 7.5.


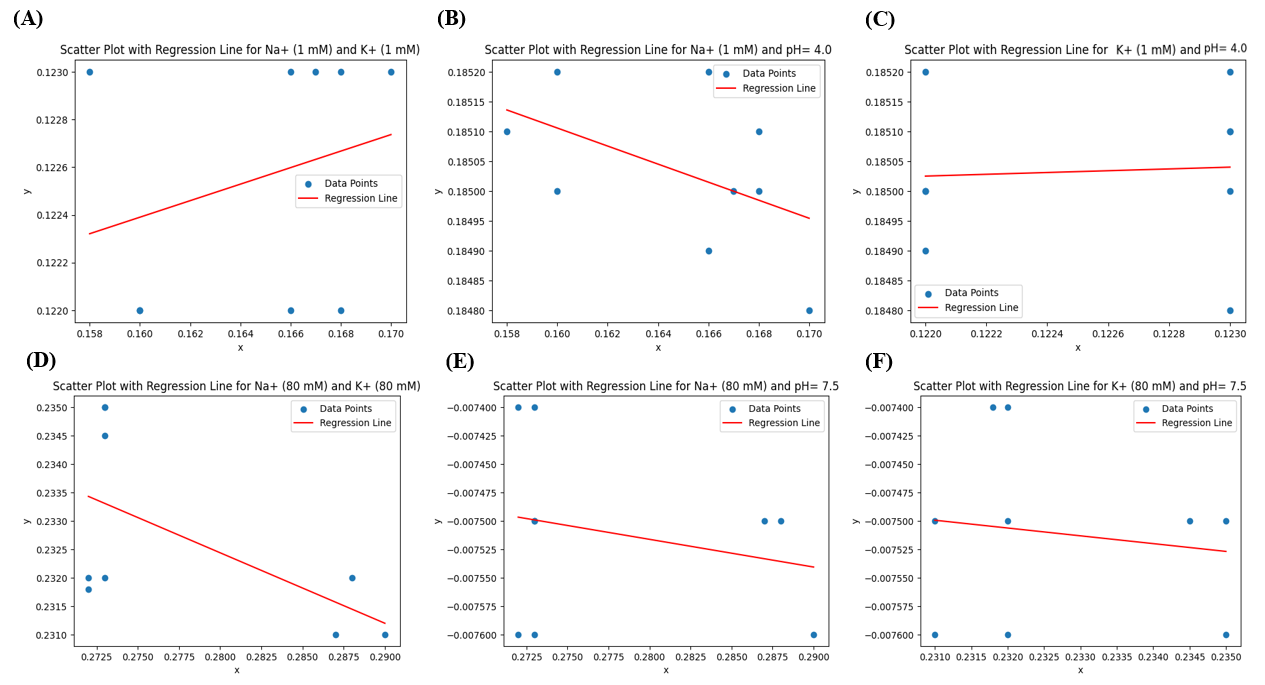


**Figure S18.** The correlation plots for the A, D) Na^+^, and K^+^ sensors, B, E) Na^+^, and pH sensors, and C, F) Na^+^, and pH sensors.


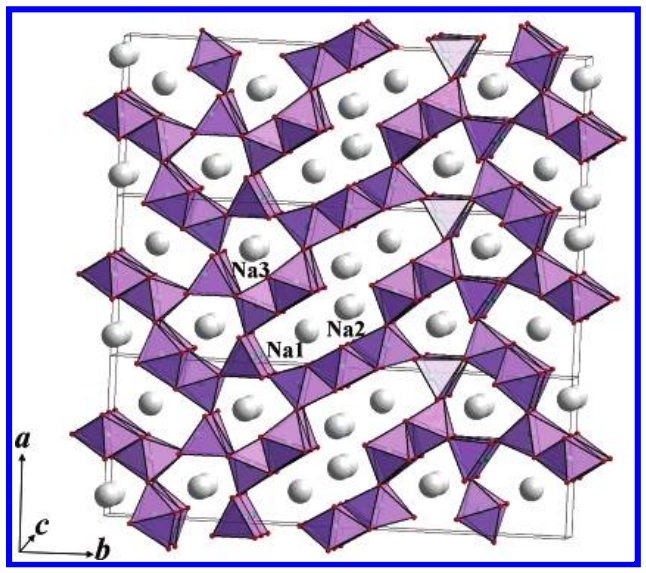


**Figure S19.** Representation of the structure of Na_0.44_MnO_2_ perpendicular to the ab plane^4^.


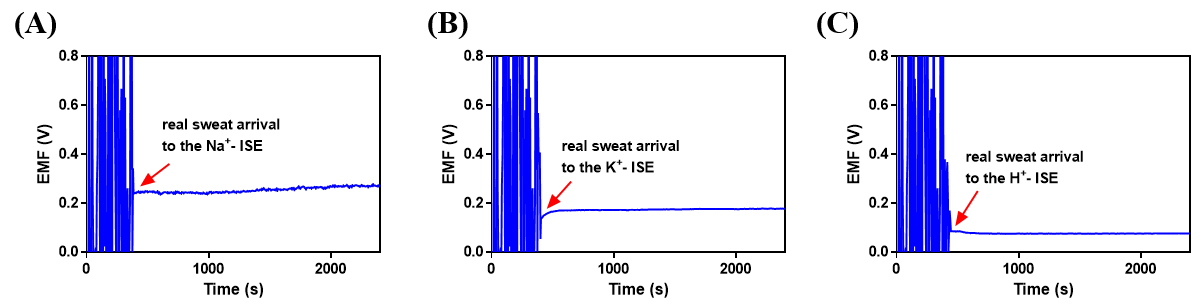


**Figure S20.** Integrated test results of the fabricated wearable potentiometric sensor for analysis of (A) Na^+^, (B) K^+^, and (C) pH in real sweat samples for volunteer 2.

**Table S1** Na^+^-selective membrane composition optimization.

| Number | Ionophore (%wt) | NaTPB (%wt) | PVC (%wt) | Plasticizer (%60) | Slope (mV/decade) |
| --- | --- | --- | --- | --- | --- |
| 1 | - | - | 40 | DBP | 31.6±0.8 |
| 2 | - | 2 | 38 | DBP | 39.8±0.9 |
| 3 | 2 | 2 | 36 | DBP | 45.7±1.2 |
| 4 | 4 | 2 | 34 | DBP | 49.8±0.6 |
| 5 | 6 | 2 | 32 | DBP | 50.2±0.4 |
| 6 | 8 | 2 | 30 | DBP | 63.4±0.8 |
| 7 | 10 | 2 | 28 | DBP | 65.6±0.6 |
| 8 | 8 | 0 | 32 | DBP | 65.3±0.8 |
| 9 | 8 | 1 | 31 | DBP | 65.4±0.9 |
| 10 | 8 | 3 | 29 | DBP | 64.8±0.9 |
| 12 | 8 | 2 | 30 | NB | 64.6±0.8 |
| 13 | 8 | 2 | 30 | DOP | 65.3±1.1 |
| 14 | 8 | 2 | 30 | OA | 64.7±1.2 |

**Table S2** K^+^-selective membrane composition optimization.

| Number | Ionophore (%wt) | NaTPB (%wt) | PVC (%wt) | Plasticizer (%60) | Slope (mV/decade) |
| --- | --- | --- | --- | --- | --- |
| 1 | - | - | 40 | DBP | 37.8±0.8 |
| 2 | - | 2 | 38 | DBP | 46.1±0.6 |
| 3 | 1 | 2 | 37 | DBP | 52.3±0.7 |
| 4 | 3 | 2 | 35 | DBP | 56.7±1.2 |
| 5 | 6 | 2 | 32 | DBP | 57.8±0.8 |
| 6 | 9 | 2 | 29 | DBP | 58.2±0.7 |
| 7 | 10 | 2 | 28 | DBP | 63.7±0.8 |
| 8 | 9 | 0 | 31 | DBP | 64.4±0.6 |
| 9 | 9 | 1 | 30 | DBP | 64.6±1.1 |
| 10 | 9 | 3 | 28 | DBP | 64.5±1.1 |
| 12 | 9 | 2 | 29 | NB | 65.2±1.2 |
| 13 | 9 | 2 | 29 | DOP | 64.4±0.9 |
| 14 | 9 | 2 | 29 | OA | 64.3±0.8 |

**Table S3** H^+^-selective membrane composition optimization.

| Number | Ionophore (mg) | Slope (mV/decade) |
| --- | --- | --- |
| 1 | 2 | -52.6±1.1 |
| 2 | 5 | -52.7±0.8 |
| 3 | 10 | -53.5±0.8 |
| 4 | 15 | -54.3±0.6 |
| 5 | 20 | -55.2±0.5 |
| 6 | 25 | -65.6±0.9 |

**Table S4** The estimated selectivity coefficients for the pH, Na^+^, and K^+^ sensors.

| Sensor | Interfering ions | Selectivity coefficient | required selectivity coefficient |
| --- | --- | --- | --- |
| pH sensor | Na^+^ | 1.4×10^-4^ | 1.0×10^-4^ |
|  | K^+^ | 1.2×10^-4^ | 4.1×10^-4^ |
|  | Ca^2+^ | 1.5×10^-4^ | 5.8×10^-4^ |
|  | Mg^2+^ | 1.4×10^-4^ | 1.2×10^-3^ |
|  | NH_4_^+^ | 1.3×10^-4^ | 1.2×10^-2^ |
| Na^+^ sensor | K^+^ | 5.5×10^-3^ | 8.3×10^-3^ |
|  | Ca^2+^ | 6.2×10^-3^ | 1.2×10^-1^ |
|  | Mg^2+^ | 5.8×10^-3^ | 2.4×10^-1^ |
|  | NH_4_^+^ | 7.9×10^-3^ | 2.5×10^-2^ |
| K^+^ sensor | Na^+^ | 3.2×10^-3^ | 4.0×10^-3^ |
|  | Ca^2+^ | 4.3×10^-3^ | 2.3×10^-2^ |
|  | Mg^2+^ | 2.0×10^-3^ | 4.7×10^-2^ |
|  | NH_4_^+^ | 3.4×10^-3^ | 5.0×10^-3^ |

**Table S5** Batch-by-batch reproducibility for three fabrication batches of pH, Na^+^, and K^+^ sensors (n = 3 electrodes).

| Sensor | Parameter | 1st batch (n=3) | | 2nd batch (n=3) | | 3rd batch (n=3) | | Interbatch | |
| --- | --- | --- | --- | --- | --- | --- | --- | --- | --- |
|  |  | Average | Std. dev. | Average | Std. dev. | Average | Std. dev. | Average | Std. dev. |
| pH | E^0^ (mV) | 397.7 | 0.5 | 412.1 | 0.8 | 380.3 | 0.9 | 396.7 | 1.3 |
|  | Slope (mV/dec) | -55.1 | 0.1 | -57.2 | 0.2 | -52.2 | 0.2 | -54.8 | 0.3 |
| Na^+^ | E^0^ (mV) | 158.3 | 0.7 | 151.7 | 0.9 | 158.6 | 1.1 | 156.2 | 1.6 |
|  | Slope (mV/dec) | 63.4 | 0.2 | 65.2 | 0.2 | 61.9 | 0.3 | 63.5 | 0.4 |
| K^+^ | E^0^ (mV) | 120.5 | 0.6 | 113.6 | 0.8 | 125.1 | 1.0 | 119.7 | 1.4 |
|  | Slope (mV/dec) | 57.9 | 0.2 | 59.3 | 0.1 | 61.3 | 0.3 | 59.5 | 0.4 |

**Table S6** Summary of sensitivity of the sensor under different ionophore amounts.

| Sensor | Ionophore | | Sensitivity (mV/decade) |
| --- | --- | --- | --- |
| Na^+^-sensor | Na_0.44_MnO_2_ | 2% (w/w) | 47.7±0.6 |
|  |  | 5% (w/w) | 54.1±0.7 |
|  |  | 8% (w/w) | 63.4±0.9 |
|  |  | 9% (w/w) | 55.6±0.8 |
|  |  | 11% (w/w) | 53.7±1.1 |
| K^+^-sensor | K_2_Co[Fe(CN)_6_] | 3% (w/w) | 48.6±1.2 |
|  |  | 6% (w/w) | 53.5±0.9 |
|  |  | 9% (w/w) | 58.2±0.7 |
|  |  | 11% (w/w) | 56.7±0.8 |
|  |  | 13% (w/w) | 51.3±1.1 |
| pH-sensor | PANI | 5 (mg) | -52.7±0.7 |
|  |  | 10 (mg) | -53.5±0.8 |
|  |  | 15 (mg) | -54.3±0.7 |
|  |  | 20 (mg) | -55.2±0.6 |
|  |  | 25 (mg) | -65.6±0.9 |

**Table S7** The estimated correlation coefficients for the pH, Na^+^, and K^+^ sensors.

| Potentiometric responses | Correlation coefficients |
| --- | --- |
| Na^+^-sensor for 1 mM Na^+^ solution and K^+^-sensor for 1 mM K^+^ solution | r = 0.28 (P-value=0.46) |
| Na^+^-sensor for 1 mM Na^+^ solution and pH-sensor for PBS with pH=4.0 | r = -0.49 (P-value=0.17) |
| K^+^-sensor for 1 mM K^+^ solution and pH-sensor for PBS with pH=4.0 | r = 0.05 (P-value=0.87) |
| Na^+^-sensor for 80 mM Na^+^ solution and K^+^-sensor for 1 mM K^+^ solution | r = -0.59 (P-value=0.09) |
| Na^+^-sensor for 80 mM Na^+^ solution and pH-sensor for PBS with pH=7.5 | r = -0.14 (P-value=0.71) |
| K^+^-sensor for 80 mM K^+^ solution and pH-sensor for PBS with pH=7.5 | r = -0.24 (P-value=0.52) |

**Table S8** Comparison of the performances of the wearable potentiometric sensors with this work.

| Analyte | Platform | Recognition  Element | Reference Electrode | Working range  (mM) | LOD  (mM) | Sensitivity  (mV/ decade) | Ref. |
| --- | --- | --- | --- | --- | --- | --- | --- |
| Na^+^ | flexible leather substrate | Na_0.44_MnO_2_ | Ag ink | 0.21-24.54 | - | 58 | 5 |
| Na^+^ | Adhesive tape | Na^+^ ionophore X | Ag/AgCl | 5 – 160 | - | 35.0 | 6 |
| K^+^ |  | valinomycin |  | 1 – 32 | - | 45.3 |  |
| Na^+^ | PDMS | Na^+^ ionophore X | Ag/AgCl/NaCl/PVB | 1 – 100 | - | 58.2 | 7 |
| K^+^ |  | valinomycin |  | 1 – 100 | - | 41.5 |  |
| H^+^ |  | PANI |  | 4 – 8 | - | 56.1 |  |
| Na^+^ | PET | Na^+^ ionophore X | Ag/AgCl ink | 5 – 100 | 1 | 51.8 | 8 |
| K^+^ |  | valinomycin |  | 1.25 – 40 | 0.5 | 31.8 |  |
| Na^+^ | PEDOT: PSS/Carbon fiber thread | Na^+^ ionophore X | Ag/AgCl ink | 0.1 – 100 | - | 60.7 ± 1.5 | 9 |
| K^+^ |  | valinomycin |  | 0.1 – 100 | - | 54.8 ± 0.6 |  |
| Na^+^ | PDMS | Na^+^ ionophore X | Ag/AgCl conductive inks | 10 – 160 | - | 60.1 | 10 |
| K^+^ |  | valinomycin |  | 2 – 32 | - | 64.5 |  |
| H^+^ |  | PANI |  | 3 – 8 | - | 60 |  |
| Na^+^ | Textile-PU | Na^+^ ionophore X | Ag/AgCl ink/NaCl + PVB/PU | 10 - 100 | 0.012 | 59.4 | 11 |
| K^+^ |  | valinomycin |  | 10 - 100 | 0.012 | 56.5 |  |
| Na^+^ | SiO_2_/Si substrates | Na^+^ ionophore X | PVC/agarose + NaCl on Ag/AgCl/CNT | 0.000708-  0.001 | 0.00316 | 56 ± 3 | 12 |
| K^+^ | PET | valinomycin | Ag/AgCl conductive ink | 0.1 - 100 | 0.12 | 58.0± 4.3 | 13 |
| Na^+^ | PVC thin sheet | Na_0.44_MnO_2_ | Ag/AgCl/PVB | 1.0-130.0 | 0.57 | 59.7 | This work |
| K^+^ |  | K_2_Co[Fe(CN)_6_] |  | 0.5-80.0 | 0.39 | 57.8 |  |
| H^+^ |  | PANI |  | 4.0-7.5 | - | 54.7 |  |

**References**

1 Abu-Thabit, N. Y. Chemical oxidative polymerization of polyaniline: A practical approach for preparation of smart conductive textiles. *Journal of Chemical Education* **93**, 1606-1611 (2016).

2 Sauvage, F., Baudrin, E. & Tarascon, J.-M. Study of the potentiometric response towards sodium ions of Na0. 44− xMnO2 for the development of selective sodium ion sensors. *Sensors and Actuators B: Chemical* **120**, 638-644 (2007).

3 Nai, J., Zhang, J. & Lou, X. W. D. Construction of single-crystalline Prussian blue analog hollow nanostructures with tailorable topologies. *Chem* **4**, 1967-1982 (2018).

4 Sauvage, F., Laffont, L., Tarascon, J.-M. & Baudrin, E. Study of the insertion/deinsertion mechanism of sodium into Na0. 44MnO2. *Inorganic chemistry* **46**, 3289-3294 (2007).

5 Ghoorchian, A. *et al.* Wearable potentiometric sensor based on Na0. 44MnO2 for non-invasive monitoring of sodium ions in sweat. *Analytical Chemistry* **94**, 2263-2270 (2022).

6 He, X. *et al.* Integrated smart janus textile bands for self-pumping sweat sampling and analysis. *ACS sensors* **5**, 1548-1554 (2020).

7 Zhai, Q. *et al.* Vertically aligned gold nanowires as stretchable and wearable epidermal ion-selective electrode for noninvasive multiplexed sweat analysis. *Analytical chemistry* **92**, 4647-4655 (2020).

8 He, W. *et al.* Integrated textile sensor patch for real-time and multiplex sweat analysis. *Science advances* **5**, eaax0649 (2019).

9 Yoon, J. H. *et al.* Extremely fast self-healable bio-based supramolecular polymer for wearable real-time sweat-monitoring sensor. *ACS applied materials & interfaces* **11**, 46165-46175 (2019).

10 Xu, G. *et al.* Battery‐free and wireless epidermal electrochemical system with all‐printed stretchable electrode array for multiplexed In situ sweat analysis. *Advanced Materials Technologies* **4**, 1800658 (2019).

11 Parrilla, M., Cánovas, R., Jeerapan, I., Andrade, F. J. & Wang, J. A textile‐based stretchable multi‐ion potentiometric sensor. *Advanced healthcare materials* **5**, 996-1001 (2016).

12 Roy, S., David-Pur, M. & Hanein, Y. Carbon nanotube-based ion selective sensors for wearable applications. *ACS Applied Materials & Interfaces* **9**, 35169-35177 (2017).

13 Sempionatto, J. R. *et al.* Eyeglasses based wireless electrolyte and metabolite sensor platform. *Lab on a Chip* **17**, 1834-1842 (2017).
